# Optogenetic screening of MCT1 activity implicates a cluster of non-steroidal anti-inflammatory drugs (NSAIDs) as inhibitors of lactate transport

**DOI:** 10.1371/journal.pone.0312492

**Published:** 2024-12-12

**Authors:** Scott A. Wegner, Hahn Kim, José L. Avalos

**Affiliations:** 1 Department of Molecular Biology, Princeton University, Princeton, New Jersey, United States of America; 2 Department of Chemistry, Princeton University, Princeton, New Jersey, United States of America; 3 Princeton University Small Molecule Screening Center, Princeton University, Princeton, New Jersey, United States of America; 4 Department of Chemical and Biological Engineering, Princeton University, Princeton, New Jersey, United States of America; 5 The Andlinger Center for Energy and the Environment, Princeton University, Princeton, New Jersey, United States of America; 6 High Meadows Environmental Institute, Princeton University, Princeton, New Jersey, United States of America; University Medical Center Göttingen, ISRAEL

## Abstract

Lactate transport plays a crucial role in the metabolism, microenvironment, and survival of cancer cells. However, current drugs targeting either MCT1 or MCT4, which traditionally mediate lactate import or efflux respectively, show limited efficacy beyond in vitro models. This limitation partly arises from the existence of both isoforms in certain tumors, however existing high-affinity MCT1/4 inhibitors are years away from human testing. Therefore, we conducted an optogenetic drug screen in *Saccharomyces cerevisiae* on a subset of the FDA-approved drug library to identify existing scaffolds that could be repurposed as monocarboxylate transporter (MCT) inhibitors. Our findings show that several existing drug classes inhibit MCT1 activity, including non-steroidal estrogens, non-steroidal anti-inflammatory drugs (NSAIDs), and natural products (in total representing approximately 1% of the total library, 78 out of 6400), with a moderate affinity (IC_50_ 1.8–21 μM). Given the well-tolerated nature of NSAIDs, and their known anticancer properties associated with COX inhibition, we chose to further investigate their MCT1 inhibition profile. The majority of NSAIDs in our screen cluster into a single large structural grouping. Moreover, this group is predominantly comprised of FDA-approved NSAIDs, with seven exhibiting moderate MCT1 inhibition. Since these molecules form a distinct structural cluster with known NSAID MCT4 inhibitors, such as diclofenac, ketoprofen, and indomethacin, we hypothesize that these newly identified inhibitors may also inhibit both transporters. Consequently, NSAIDs as a class, and piroxicam specifically (IC_50_ 4.4 μM), demonstrate MCT1 inhibition at theoretically relevant human dosages, suggesting immediate potential for standalone MCT inhibition or combined anticancer therapy.

## Introduction

The classic metabolic phenotype of cancer cells is the Warburg effect, characterized by a preference for anaerobic lactate fermentation [1]. Lactate plays a pivotal role as both a metabolic and signaling molecule within the tumor microenvironment, providing an energy source for aerobic cancer cells, and contributes to immunosuppression. Consequently, inhibition of lactate transport presents an attractive therapeutic approach to disrupt tumor growth, enhance immune response, and improve the effectiveness of existing treatments [2]. The SLC16A monocarboxylate transporter subfamily (MCTs, particularly MCT1/4) are responsible for the proton-linked symport of lactate across the plasma membrane, which is critical for cancer cell survival [3]. The significance of these transporters is reflected in MCTs being clinical prognostic markers, with MCT induction related to disease progression and MCT expression level negatively correlating with overall disease survivability for several forms of cancer [4].

Interest in MCT inhibition has led to the development of several MCT1 inhibitors (AZD3965/BAY8002/SR13800) with high affinity and specificity towards MCT1 but with minimal activity towards MCT4 [5,6]. AZD3965 generated substantial interest, with numerous preclinical in vivo xenograft studies yielding a positive treatment outcome [7]. In MCT1-expressing cancer cell models, AZD3965 treatment consistently inhibits tumor growth, and several studies report increased chemosensitivity or radiosensitivity [7]. The success of this compound led to a highly anticipated phase-1 clinical trial in patients with advanced cancer, where AZD3965 was found to be relatively well tolerated, with dose-limiting toxicity relating to retinopathy at higher AZD3965 concentrations [8]. However, extension of this study failed to show a significant impact on cancer due to a large attrition rate (only 1 of 3 remaining subjects exhibited full remission). While MCT1 inhibition is effective in specific MCT1-dependent cancer models, cancer cells often do not rely solely on the activity of a single MCT transporter. Instead, tumors often have a mixed metabolic phenotype with MCT4 expressing cells fermenting, and MCT1 aerobically catabolizing, lactic acid. Thus, a drug which inhibits both major isoforms (MCT1 and MCT4) may present a more promising strategy.

This premise is supported by research involving the anti-hypertensive syrosingopine, which, amongst its other actions, is described as an MCT1/4 inhibitor. Importantly, 10 μM syrosingopine was found to inhibit in vitro cancer cell growth when administered alone; however, for in vivo cancer models syrosingopine was only effective in reducing tumor growth when paired with the mitochondrial oxidative phosphorylation inhibitor metformin [9,10]. Although promising for combined treatment therapies, syrosingopine has somewhat weak activity towards MCT1/4, as the dose required to inhibit tumor growth in mice (7.5 mg/kg) far exceeds tolerated human doses (~0.5 mg/kg) and is three times higher than an amnestic rodent dosage [11–13]. Thus, there is a strong motivation to develop more potent inhibitors, which was realized in recent years with a series of cyanocinnamic acid derivatives (a-CHC) with low nanomolar potency [14–16]. However, these derivatives have poor drug-like qualities and a-CHC is known to have off-target activity [17,18]. Overall, there is significant potential for the discovery of new MCT1/4 inhibitors for cancer inhibition due to the prognostic relevance of MCT1/4 expression, the results and drawbacks of syrosingopine, and the current absence of a well-validated high-affinity MCT1/4 inhibitor [19].

Here we screened a subset of the FDA-approved drug library to repurpose drugs towards MCT inhibition, utilizing a novel optogenetic screening method in *Saccharomyces cerevisiae*. The heterologous *Rattus norvegicus* MCT1 transporter was functionalized in yeast, and converted into a mevalonate transporter, using a previously published mutation [20]. This allows growth control using our optogenetic mevalonate auxotroph (optoMEV), with growth rescue mediated by MCT1 import of mevalonate. This allows a biphasic screening approach: in the first non-permissive phase of the screen MCT1-specific and non-specific compounds inhibit yeast growth, which is then rescued for specific inhibitors by blue light, rendering MCT1 activity dispensable for growth. This led us to identify a molecular cluster enriched for natural products, which display known nonselective MCT inhibition, and contains several NSAIDs likely to display similar activity. Notably, previous studies have indicated that certain NSAIDs affect in vitro lactate transport, with diclofenac, one of our exemplars, previously shown to be a nonspecific MCT1/4 inhibitor [21]. Further screening of additional NSAIDs revealed that many current FDA-approved NSAIDs (7 out of the 14 chosen for screening) exhibit moderate levels of MCT1 inhibition without off-target growth inhibition at relevant concentrations. These findings underscore the potential of NSAIDs as a readily available class of compounds for drug repurposing as MCT inhibitors.

## Results

### OptoMEV: Optogenetic control of mevalonate production

**Fig 1:**
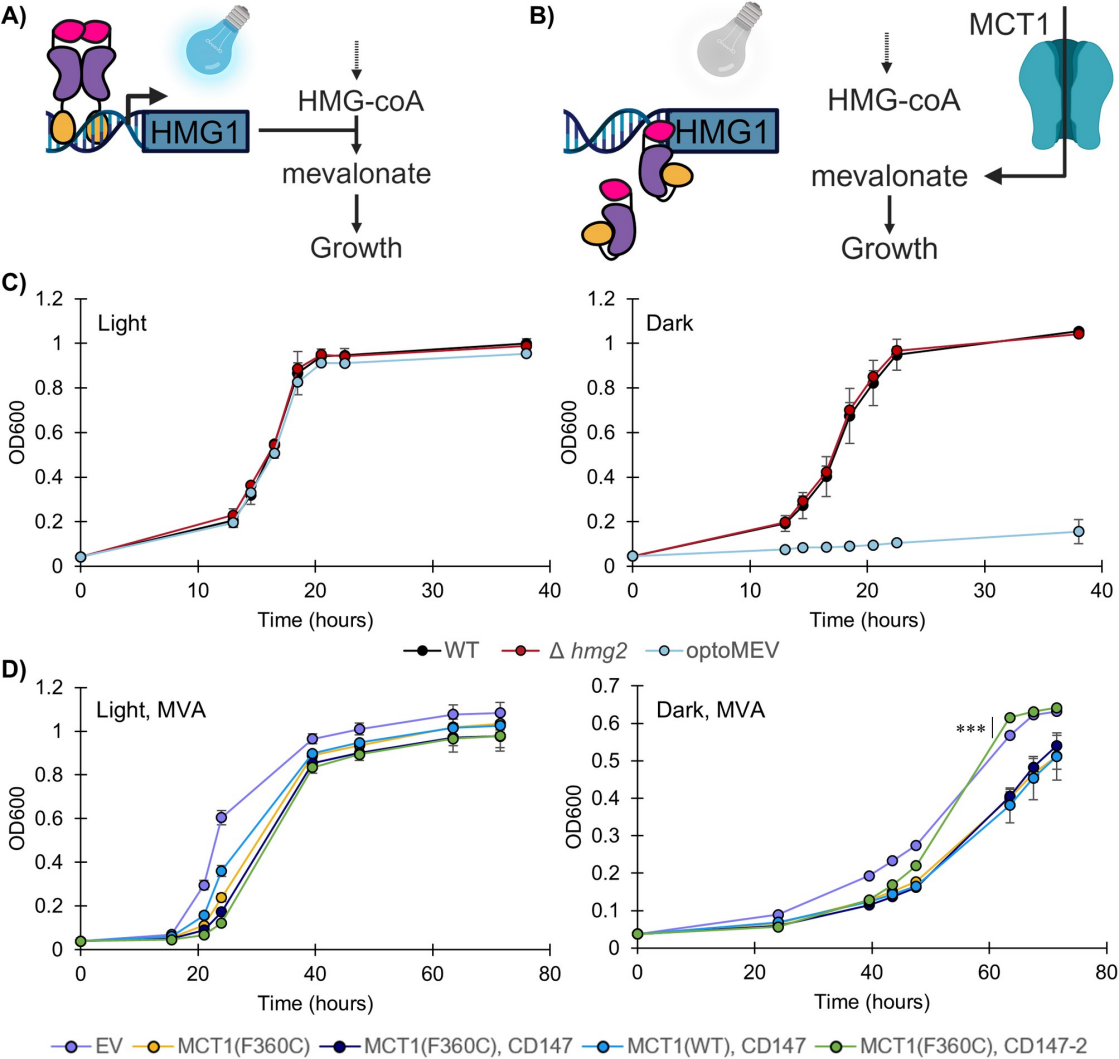
Characterization of optoMEV and mevalonate transport by the unmodified *Rattus norvegicus* MCT1. **A-B)** Optogenetic regulation of *HMG1* expression using the blue light-sensitive EL222-VP16 transcription factor allows for transcription of HMG1 from the pC120 promoter under permissive light conditions. **C)** In permissive conditions (Light) all strains are able to grow. In contrast, in non-permissive (Dark) conditions, the optoMEV strain, which has a deletion of the *hmg1* paralog *hmg2*, is unable to grow as *hmg1* controls the production of mevalonate, an essential molecule in *S*. *cerevisiae*. Addition of MCT1 provides a means to utilize extracellular mevalonate and recover growth. **D)** Expression of the *Rattus norvegicus* MCT1 protein in conjunction with its accessory protein CD147 from a high-copy 2μ plasmid. Cells were cultured in SC-URA media (pH 5) supplemented with 10 mM mevalonate (MVA). Graphs depict the results from biological quadruplicates, with mean growth plotted for each time point and error bars representing standard deviation. Statistical analysis performed using Student’s t-test, *** p < 0.001.

To introduce dependence upon MCT1 activity, we employ an optogenetic strain that conditionally produces the essential metabolite mevalonate. This strain (optoMEV; SAWy524) uses the blue light-responsive transcription factor EL222-VP16 to control expression of *HMG1* from the pC120 promoter (Fig 1A and 1B). Permissive blue light conditions allow for full growth recovery comparable to the non-optogenetic parent strains (Fig 1C, left). However, in dark non-permissive conditions there is a strong decrease in the growth rate of the optoMEV strain, reflecting 12% that of the parent strain (Fig 1B, right; S1 Table). Furthermore, we have previously characterized that this strain is insensitive to mevalonate supplementation in the media, necessitating MCT1 activity to recover growth (Wegner SA, Jiang V, Cortez JD, Avalos JL. [In preparation]).

The *R*. *norvegicus* MCT1 transporter which exhibits reported function in yeast, with the F360C mutation which confers mevalonate recognition, was expressed with its accessory protein CD147 (also known as basigin) [20,22]. Contrary to previous reports with high-copy 2μ MCT1 expression in yeast [22], we observe a drop in the permissive growth rate with MCT1 expression (Fig 1C, left; S2 Table). This growth deficit appears to be partially due to the F360C mutation, as the growth rate of an equivalent wild-type MCT1 construct is considerably higher. Despite this, expression of MCT1(F360C) with CD147-2 results in slightly earlier OD600 saturation at 63.5 hours compared to the empty vector in non-permissive conditions with mevalonate feeding (Fig 1C, right). Thus, there is evidence for mevalonate-dependent growth, although it suggests that the heterologous MCT1 protein is poorly functionalized in *S*. *cerevisiae*.

### Established secretion tags support MCT1 localization and activity

**Fig 2:**
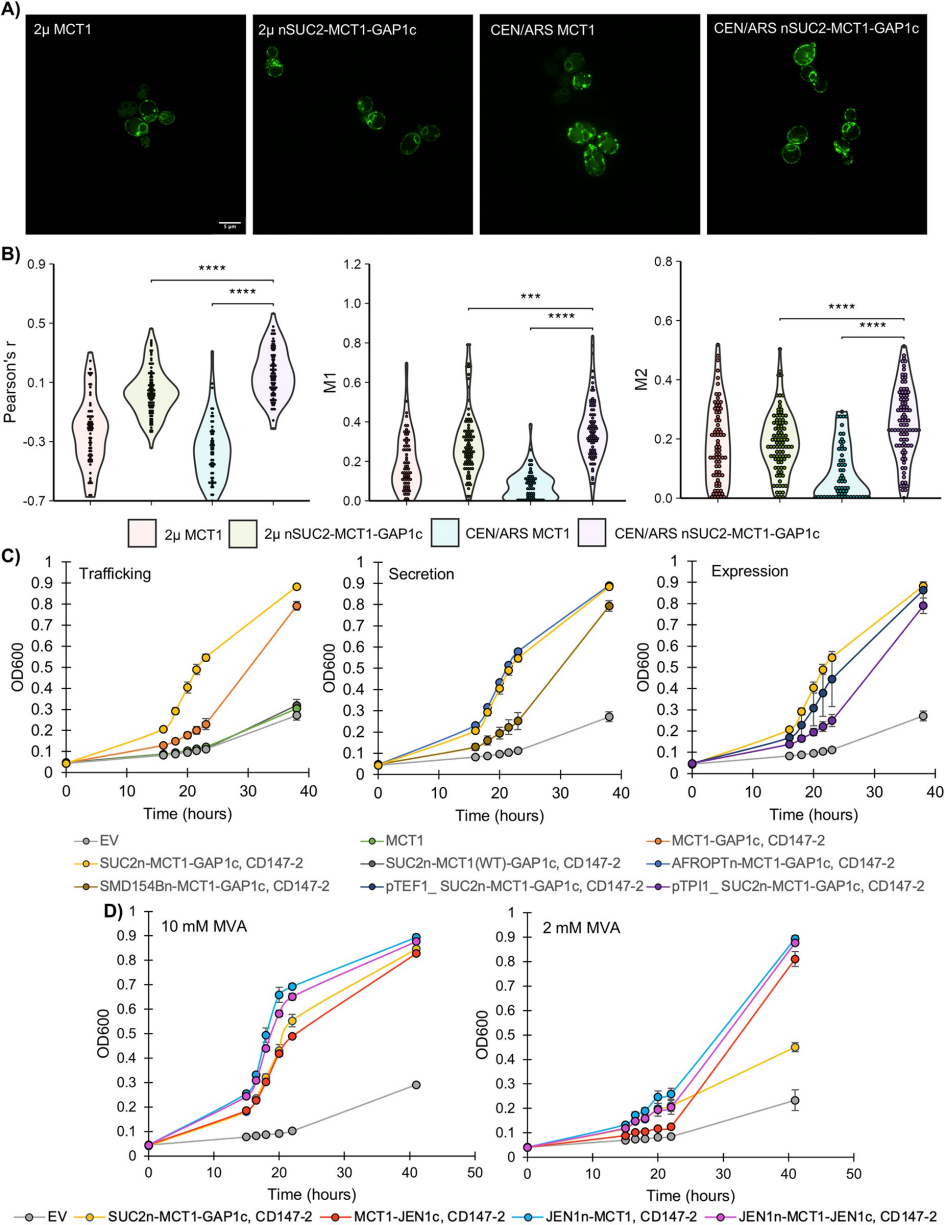
Optimization of MCT1 function in *S*. *cerevisiae* yields a highly functional mevalonate-transporting protein chimera. **A)** Comparison of the optimal tagged construct for expression using either a high-copy 2μ vector or a low-copy CEN/ARS vector, relative to untagged MCT1. The MCT-GFP fusion was constructed with a c-terminal linkage as described in Methods. The scale bar is 5 μm. **B)** Quantification of plasma membrane overlap (not shown) with the MCT1-GFP signal in **(A)**. The correlation (Pearson’s r) or degree of overlap (Manders M1/M2) between the MCT1 signal and the plasma membrane was quantified for n = 50 cells from each construct. **C)** Comparison of mevalonate-dependent growth (SC-URA, pH 5, 10 mM mevalonate) in non-permissive conditions exploring the contribution of either the c-terminal trafficking tag (Fig 2C, left), different sources of secretory tags (middle), and different promoter expression strengths (right). The order of promoter strength goes from pGPD1 (the promoter used when not specified) > pTEF1 > pTPI1. **D)** Mevalonate-dependent growth of a series of N/C-terminal chimeric fusions of MCT1 to JEN1 domains related to plasma membrane trafficking (see associated S4 Fig for alternative chimeric truncations). These chimeric transporters are compared for growth relative to the optimal tagged transporter variant (SUC2n-MCT1-GAP1c; yellow) for growth in either 10 mM mevalonate (left) or 2 mM mevalonate (right). All MCT1 constructs, where not specifically denoted as wild type (WT), bear the F360C mutation which allows recognition of mevalonate. The growth curves in **(B-D)** represent biological quadruplicates, where mean growth is plotted for each time point and error bars represent standard deviation. OD600 is reported in Tecan units. Statistical significance: *** p < 0.001, **** p < 0.0001.

The inability of MCT1 to recover growth likely results from mislocalization, as evidenced by the retention of a MCT1-GFP fusion to the endoplasmic reticulum (S1 Fig) [22]. To improve the trafficking of MCT1 we first introduced established tags, known to promote either protein secretion or targeting to the plasma membrane. These modifications were verified both for localization and functional monocarboxylate transport, through growth in pyruvate or lactate as a sole carbon source, which serves as a robust selection condition. Addition of a c-terminal plasma membrane association module from *GAP1* (GAP1c), mitigates ER retention and increases apparent localization to the plasma membrane (S2 Fig) [23]. However, this is not associated with an increased growth in pyruvate or lactate relative to the empty vector (S2 Fig). In contrast, the further addition of an n-terminal secretion tag (SUC2n) yields a construct that not only associates with the plasma membrane (S3 Fig) but also results in a higher growth rate in both lactate relative to empty vector (S3 Fig; S3 Table). As a final optimization, SUC2-MCT1-GAP1c was transferred to a low-copy CEN/ARS resulting in even higher levels of membrane association (Fig 2A and 2B).

To ensure the applicability of these optimizations to mevalonate transport, the tagged constructs were screened using the optoMEV strain. To first demonstrate that observed growth was due to MCT1 activity, the wildtype MCT1 sequence was compared to the F360C mutant for equivalent constructs, with growth only observed with the mevalonate recognizing mutant (Fig 2C, left; S4 Table). Next, the c-terminal tag was compared to the n/c-terminal construct, which again demonstrates the importance of the n-terminal tag to support MCT1 localization and mevalonate-dependent growth. Despite the importance of an n-terminal secretion tag for membrane targeting, it’s noteworthy that not all tags appear to be equivalent. For instance, the synthetic SMD154n tag fails to yield equivalent growth relative to either SUC2n or an optimized alpha factor leader sequence (AFROPTn; Fig 2C, middle) [24,25]. The SUC2n-MTC1-GAP1c construct using the strong pGPD1 promoter provides the highest mevalonate-dependent growth rate of 0.158 ± 0.006 hr^-1^, which is 67% that of the optoMEV parent strain in permissive conditions (Fig 2C, right; S4 Table). Thus, this dual tagged construct exhibits both high levels of plasma membrane association and confers robust mevalonate-dependent growth.

### MCT1-JEN1 chimeric transporters outperform established secretion tags

To explore whether MCT1-dependent growth could be further enhanced, a series of MCT1/*JEN1* chimeric transporters were tested. Domains within the cytosolic termini of *JEN1* have been previously shown to support membrane localization, without conferring glucose-mediated receptor internalization traditionally exhibited by this transporter (JEN1n - residues 95–133, JEN1c –residues 555–583), and were used to guide localization of MCT1 (S4 Fig) [26]. Based on mevalonate-dependent growth it was found that the most functional fusion construct is one in which the *JEN1* terminal domains are added to the full length MCT1 protein, as many of the truncations result in lower or no growth (S4 Fig). These results indicate that despite not supporting membrane association in yeast, the cytosolic termini of MCT1 are critical for either structural stability or function.

Given the substantial impact of the N-terminal SUC2 tag on previous construct optimizations, we investigated the effect of adding each *JEN1* domain separately. Interestingly, either domain was sufficient to improve growth, with the N-terminal *JEN1* chimera achieving a growth rate of 0.195 ± 0.008 hr^-1^, surpassing that of the SUC2n-MCT1-GAP1c construct (Fig 2D, left; S5 Table). Even under challenging conditions (2 mM MVA) the growth rate of the n-terminal *JEN1* chimera is higher than either the c-terminal chimera, the n/c-terminal dual chimera, or the SUC2n-MCT1-GAP1c tagged construct (SAWy741 referred to as optoMEV-MCT1). Furthermore, in challenging conditions the chimeras achieve a much higher endpoint saturation compared to the tagged construct (Fig 2D, right). Thus, while both strategies are effective, the *JEN1* n-terminal chimera shows the highest overall degree of activity.

### MCT1 inhibition by AZD3965

**Fig 3:**
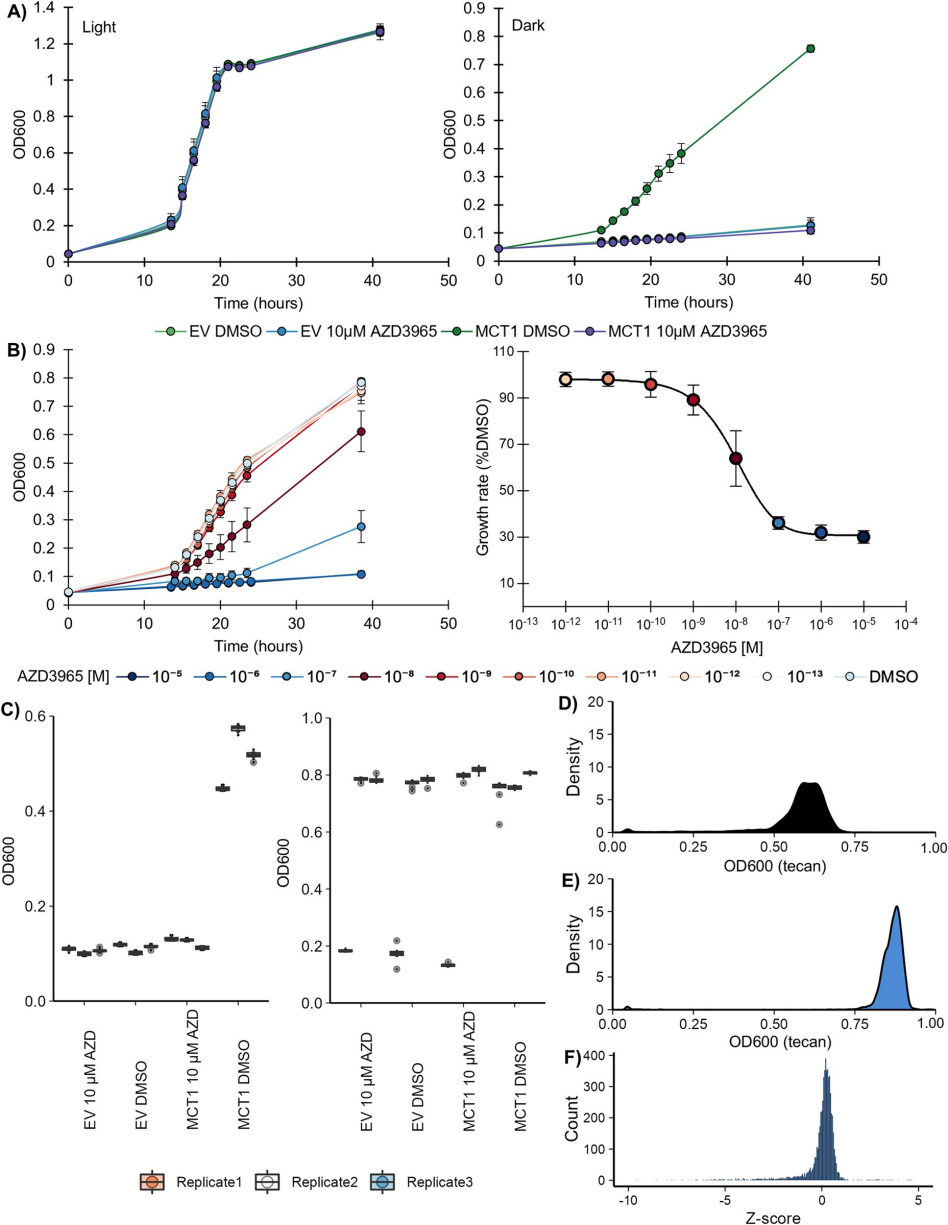
The optoMEV-MCT1 growth assay returns relevant IC_50_ parameters for the known inhibitor AZD3965 and allows screening of an FDA-approved library of compounds. **A)** Sensitivity of the optoMEV-MCT1 (MCT1; SAWy741) strain to the well-validated inhibitor AZD3965 was assayed under both light (permissive; left) and dark (non-permissive; right) conditions relative to the optoMEV-EV (EV) strain containing an empty CEN/ARS vector. Under permissive conditions, the activity of MCT1 in this strain is dispensable, and growth is insensitive to the presence of AZD3965. **B)** Calculation of IC_50_ concentrations for MCT1 mevalonate-dependent growth shown as raw OD600 Tecan unit readings over time (left) and the specific growth rate relative to each drug dosage (Right). **C)** High-throughput validation of the MCT1-dependent growth assay using the same inhibitor and strains as above. Triplicate independent experiments, performed separately from unique inoculations with individual media preparations, were assayed for AZD3965 (AZD) inhibition relative to vehicle treatment (DMSO). For the first 24 hours, cells were grown in non-permissive conditions, following which two replicates were transferred to permissive conditions for 24 hours (Replicate 1 was left in non-permissive conditions for 48 hours total). For each condition and each replicate, the average is plotted, with individual data points (n = 10) shown. **D-F)** High throughput screening of the drug library using the optoMEV-MCT1 strain and OD600 following growth in 24 hours non-permissive conditions **(D)**, followed by permissive conditions **(E)**, which was converted into a growth fraction and normalized by plate to reflect a Z-score distribution shown in **(F)**.

To evaluate whether our optogenetic MCT1 strain was suitable for screening MCT1 inhibitors we first compared drug sensitivity to the well characterized and specific inhibitor AZD3965. For our optogenetic method to be successful AZD3965 should only inhibit under non-permissive dark conditions, where mevalonate import is necessary for yeast growth, and not under blue light. Additionally, as AZD3965 is highly specific for MCT1, we chose to screen a dose that is approximately 1000 times higher than its IC_50_ to ensure that our strains do not exhibit any general drug susceptibility. The optoMEV-MCT1 strain behaves as anticipated, with no difference between vehicle (DMSO) or AZD3965 administration in permissive conditions (Fig 3A, left; S6 Table). In contrast, only the DMSO treated optoMEV-MCT1 strain is able grow under non-permissive conditions demonstrating that AZD3965 administration is effective at abolishing mevalonate-dependent growth (Fig 3A, right). To confirm that optoMEV-MCT1 could return pharmacologically relevant parameters, we determined the IC_50_ for growth inhibition (Fig 3B). Fitting the dose-response yields an IC_50_ of 10 nM, which is in line with a previously reported IC_50_ value of 5.12 nM, indicating that our optoMEV-MCT1 strain is an effective quantitative assay of MCT1 activity [27].

To demonstrate the functional equivalence between the wild-type and mutant MCT1 (F360C) protein, AZD3965 inhibition of lactate transport was assessed. A *S*. *cerevisiae* strain absent the ability to transport lactate (by4741 *Δjen1*) was only able to recover growth, in media with lactate as the sole carbon source, with MCT1 expression (S5 Fig). AZD3965 administration inhibited growth for both forms of MCT1 (wildtype and F360C), dropping growth levels to that of the empty vector, while vehicle administration had no effect. Not only does this demonstrate the functional equivalence between assay formats, but also between MCT1 protein orthologs as AZD3965 exhibits MCT1 inhibition in both rodents and human cell lines [7,28]. Therefore, compounds which inhibit mevalonate transport of the mutant *R*. *norvegicus* MCT1 transporter should also inhibit wildtype MCT1 lactate transport in humans.

### MCT1 mevalonate assay high-throughput optimization

To facilitate library screening it was necessary to scale-down the mevalonate assay to a suitable high-throughput format. We assessed AZD3965 sensitivity in both 96-well and 384-well formats to determine which provides the best signal-to-noise ratio (S6 Fig). The 384-well non-shaking 50 μl assay condition was selected based on fold-change between conditions and was subsequently tested for assay robustness. The Z-factor was characterized from three independent experiments using DMSO and AZD3965 as negative and positive controls with an average Z’ of 0.921 ± 0.013 which is indicative of an ideal assay (S7 Fig) [29].

To demonstrate that this new scale was appropriate for our intended optogenetic drug screen we conducted triplicate experiments where, for two replicates, the replicates were transferred to permissive conditions following non-permissive incubation, while the final replicate remained in darkness throughout (Fig 3C). This was done to mimic our screen, where specificity testing would be determined in two phases: in the first non-permissive phase of the screen MCT1-specific and non-specific compounds inhibit yeast growth, shifting to blue light conditions renders MCT1 activity dispensable and allows growth recovery for specific inhibitors. As expected, exposure to blue light successfully rescues the growth of AZD3965-treated cells, whereas drug treated cells kept in continuous darkness do not recover growth throughout the 48-hour period. Thus, even with prolonged incubation AZD3965 treated cells do not recover growth, unless there is a shift to blue-light conditions.

### FDA approved and biologically annotated library screening

To discover new compounds with activity towards MCT1 we performed a biphasic screen on a molecular library of ~6400 molecules comprising a subset of the FDA-approved library as well as other molecules with annotated biological activity, using AZD3965 as a reference (S8 Fig). The growth fraction in permissive versus non-permissive conditions was used to normalize data to determine specific inhibitors: compounds of interest should have a low fraction, while inactive or non-specific compounds would exhibit a higher fraction (Fig 3D and 3E). Overall, the library itself exhibits a fairly normal distribution, with an enrichment in the left-tail indicating potential MCT1 inhibitors (Fig 3F). Among these compounds only those with a Z-score of -3.0 or lower were considered significant hits (154 compounds, 2.4% of the total library) and subjected to further analysis.

**Fig 4:**
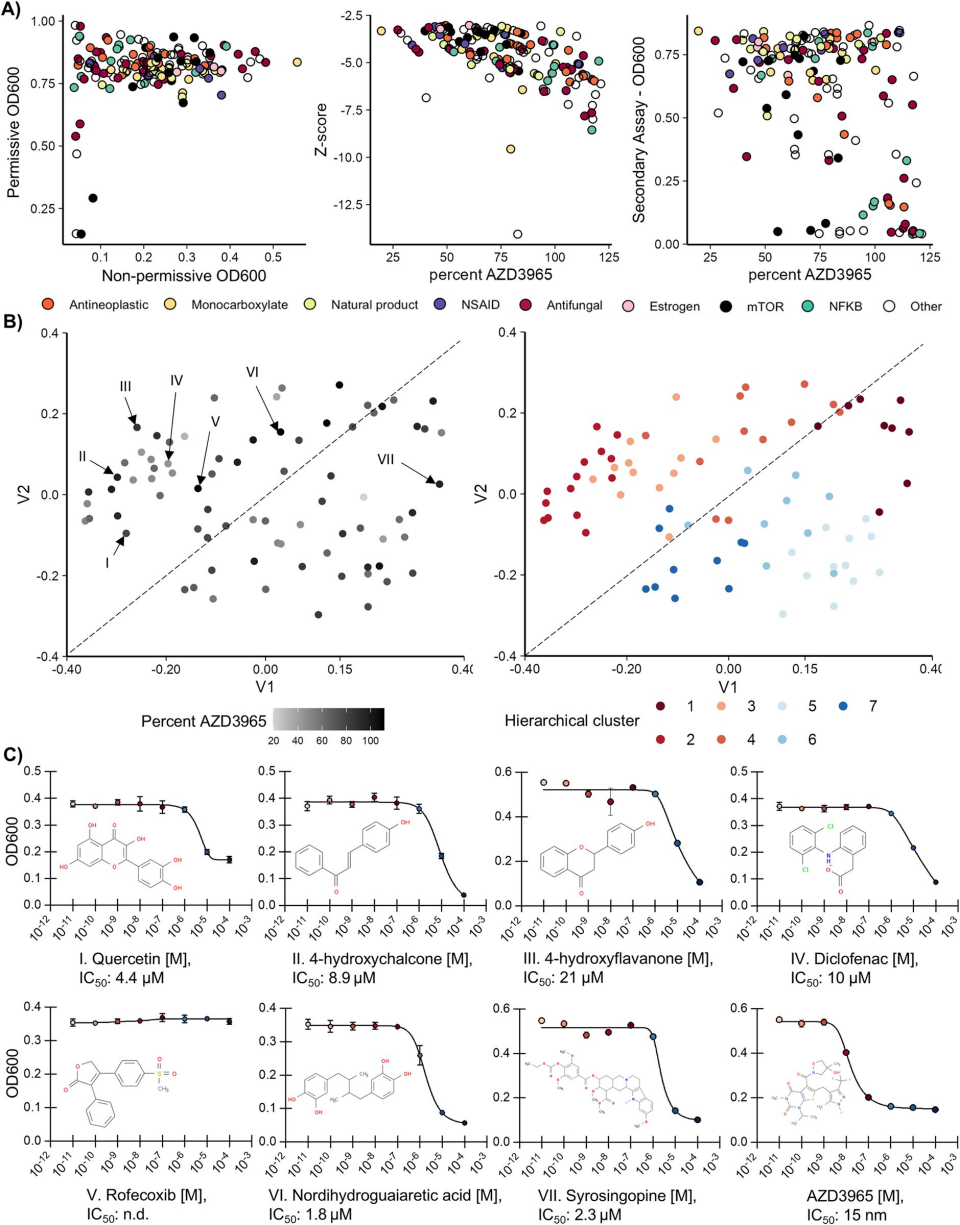
Library validation, molecular clustering, and rescreening of potential non-selective MCT inhibitors. **A)** The 154 drug candidates were compared for overall assay growth (Left), inhibitor efficacy relative to the exemplar AZD3965 (middle), and off-target cytotoxicity (right). The off-target assay was performed separately on all 154 candidates using the non-optogenetic parent strain (SAWy518), lacking MCT1 expression. Cells were grown for 18 hours prior to OD measurement. Fill represents manual compound grouping based on compound class or target. OD600 is reported in Tecan units. **B)** Multi-dimensional scaling of all positive hits that were identified in the rescreen as lacking off-target activity (n = 78), with coloring for either percent AZD3965 efficacy or hierarchical molecular cluster. Roman numerals reflect drugs tested in **(C)**, while the dotted lines represent the categorization of compounds as more (top-left) or less similar (bottom-right) to known non-selective MCT inhibitors. **C)** IC_50_ determination of the compounds identified as known, related to, or putative MCT inhibitors identified in **(B)**. The mean growth of biological quadruplicates after 18 hours of incubation is shown, with error bars representing standard deviation. IC_50_ values for each compound are shown using the best fit of a non-linear regression, except where not possible to be determined (n.d.).

To gain insights into these identified compounds, putative hits were first manually grouped into crude biological function, molecular target, or compound classification. The most common groupings being antifungals (25 molecules), antineoplastics (16), natural plant products (17), inhibitors relating to the mTOR pathway (14), NF-kβ inhibitors (10), NSAID COX inhibitors (7), and estrogen receptor agonists (6), while the remaining compounds did not have a clear functional grouping. Although there are no clear trends when analyzing non-permissive versus permissive growth (Fig 4A, left), group differences emerge when comparing the ability to inhibit MCT1 relative to the established inhibitor AZD3965 (Fig 4A, middle). NF-kβ inhibitors and antineoplastics appear to strongly inhibit MCT1 with 7/10 compounds within 90% AZD3965 efficacy and 12/16 within 75% respectively for each group. In contrast, estrogen receptor agonists (non-steroidal estrogens) appear to weakly inhibit MCT1 with all group members falling below 75% of AZD3965 inhibition. The remaining groups lack a clear distribution in terms of MCT1 inhibition efficiency.

One particular concern is the sizable fraction of compounds (32/154) that appear to be even more potent, based on growth inhibition in non-permissive conditions, than the nanomolar active inhibitor AZD3965. One potential explanation would be off-target activity; however, virtually all the tested compounds fully recover in permissive conditions, indicating on-target activity (Fig 4A, left). Another potential explanation is compound degradation–a strong non-specific growth inhibitor which degrades over time could yield a potential false-positive. To address this, the non-optogenetic parent strain (SAWy518) without dependence on MCT1 activity was rescreened with the 154-member sub-library. Upon rescreening approximately half compounds do indeed appear specific for MCT1 inhibition, with 51.3% (78/154) of the compounds within 10% the average OD600 from untreated wells after 18.5 hours of growth (S9 Fig). However, by 48 hours the number of growth inhibitory compounds decreases, as evidenced by the strong correlation between the 18.5- and 24-hour OD600 values (r^2^ = 0.956) and the lower correlation at 42 hours with most compounds eventually recovering growth, as was observed in the primary screen (S9 Fig; r^2^ = 0.589). As the secondary screen was performed in the same media as the primary screen, without exposure to light, it appears that off-target compounds are generally unstable with prolonged incubation.

From the results of the primary and secondary screen we demonstrate on-target activity for 78 compounds. To further analyze the molecular diversity of these putative MCT1 inhibitors, molecules were broken into hierarchical family clusters and potted with multidimensional scaling (Fig 4B; S10 Fig). Cluster two, composed of chalcones and flavonoids, contains the largest number of known nonselective MCT inhibitors. For instance, quercetin (a flavonoid) and phloretin (a chalcone) are classical weak MCT1/4 inhibitors and are both within this molecular cluster [17]. We used this cluster to determine which molecules resemble known non-selective inhibitors, of which an NSAID containing cluster and a non-steroidal estrogen-like cluster exhibit the closest similarity based on proximity. Seven candidate molecules, including candidates from the three identified clusters, and syrosingopine (a known MCT1/4 inhibitor), were obtained from chemical suppliers and assayed for activity versus MCT1. Every rescreened molecule except for rofecoxib exhibits activity towards MCT1 with similar IC_50_ values in the low micromolar range (Fig 4C). Thus, although no molecule approaches the affinity of AZD3965 (IC_50_ of 15 nM in the 384-well format), many FDA-approved and drug-like molecules are inhibitors of MCT1.

### FDA approved NSAIDs are inhibitors of MCT1 activity

**Fig 5:**
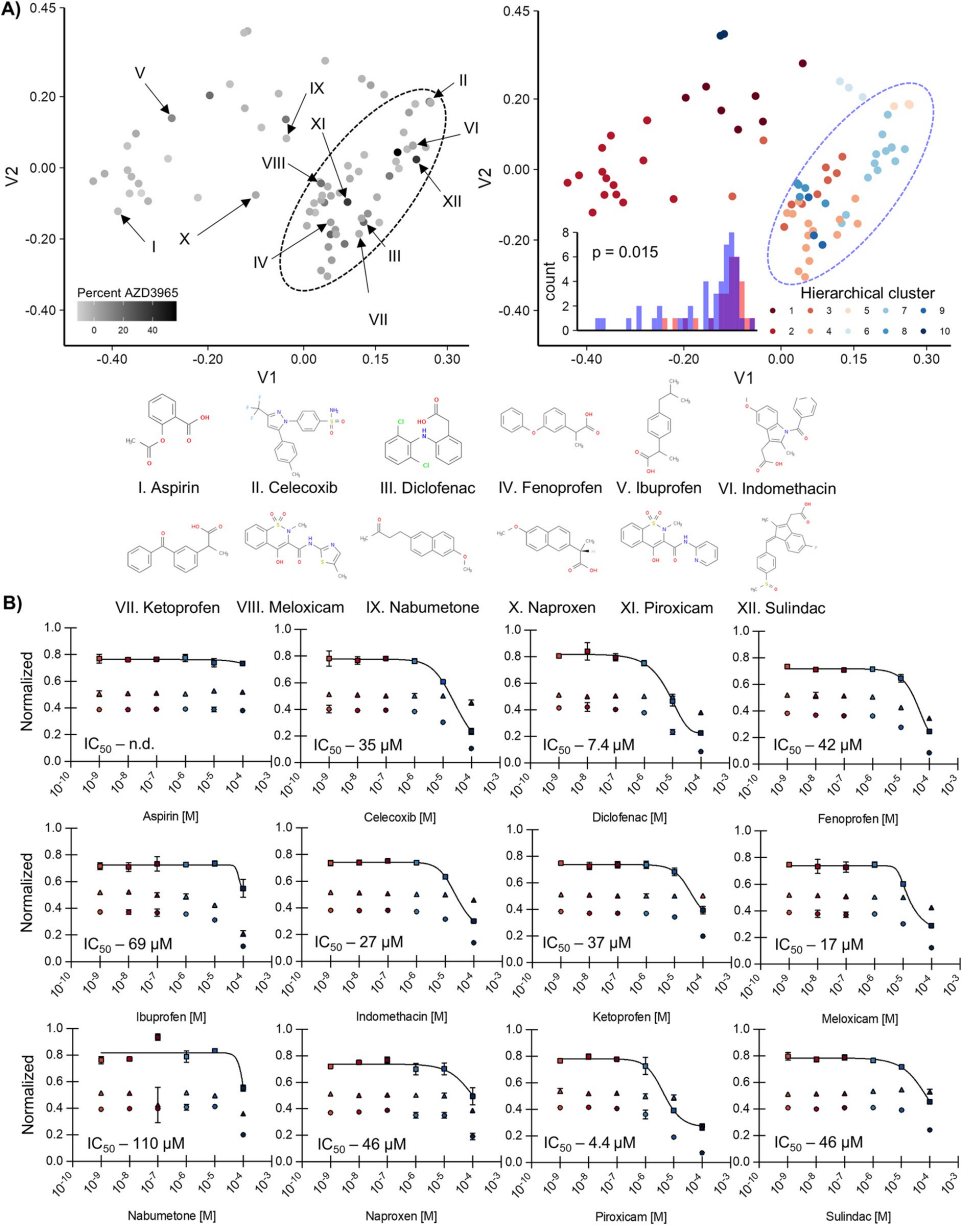
Testing FDA-approved NSAID inhibitors demonstrates that seven are relevant inhibitors of MCT1 activity. **A)** Multi-dimensional scaling of all NSAID compounds tested in the original screen (annotated as having COX inhibition), with coloring for either percent AZD3965 efficacy or hierarchical molecular cluster. The dotted ellipse represents a cluster observed manually, with verification of enrichment performed by t-test on the percent AZD3965 inhibition of the described cluster (red bars) versus all other compounds (blue bars; right, insert). Roman numerals reflect 12 of the 20 FDA-approved NSAID compounds both within and outside (aspirin, ibuprofen, nabumetone, naproxen) the observed cluster, with the structures of each molecule shown below the graph. **B)** IC_50_ determination of the NSAIDs identified in **(A)**. Growth at each concentration of the tested inhibitor of the optoMEV-MCT1 construct (circles) versus the wildtype parent strain (SAWy518; triangles) was used to normalize the drug dose-response (squares) and control for off-target activity. The mean growth of biological quadruplicates after 18 hours of incubation is shown, with error bars representing standard deviation. IC_50_ values for each compound are shown from the normalization using the best fit of a non-linear regression, except where not possible to be determined (n.d.).

We chose to further explore the potential of the NSAID class due to previous reports that NSAIDs have known anti-cancer activity and certain members inhibit MCT4 [30]. As a first step we performed hierarchical clustering on all putative NSAIDs tested in the preliminary screen, based on designation as a COX1/2 inhibitor, resulting in a list of 76 molecules. Clustering identified a superfamily composed of 6/10 of the identified groupings (Fig 5A—groups 3–5 and 7–9; S11 Fig), which is enriched for MCT1 inhibitors (Fig 5A, right insert). Additionally, many of the FDA-approved NSAIDs are within in this cluster (15/20; with the exceptions being Aspirin, Etodolac, Ibuprofen, Nabumetone, Naproxen). Therefore, we chose to analyze compounds from this cluster for their ability to inhibit MCT1.

All tested NSAIDs within the identified superfamily inhibit MCT1 with IC_50_ values ranging from 4.4 (piroxicam) to 46 μM (sulindac; Fig 5B). Importantly, the previously reported inhibition value for MCT1 by diclofenac is relatively similar to that determined by our assay (IC_50_ 1.4 μM versus 7.4 μM) [21]. Members within the identified cluster also have significantly higher potency for MCT1 inhibition relative to those outside, even with the conservative assumptions that compounds without observable activity have an IC_50_ of 100 μM (t-test, p = 0.002). Within this cluster are previously confirmed MCT4 inhibitors whose rank efficiency for MCT1 we can now compare–diclofenac appears to be the strongest MCT1 inhibitor (7.4 IC_50_ μM), followed by indomethacin (27), and ketoprofen (37). Thus, several of the currently FDA approved NSAIDs also exhibit cross-reactivity towards MCT1, making them strong MCT inhibitor candidates.

## Discussion

Using a novel optogenetic screen, where both MCT1 inhibition and specificity testing could be determined in a single screen through the use of a conditional mevalonate auxotrophy, we demonstrated MCT1 engagement for potential drug repurposing of a wide range of established chemical compounds. From an initial library of 6400 candidates comprising FDA approved drugs and biologically annotated molecules we discovered 78 molecules which display moderate activity towards MCT1. This final list was composed of antifungals (8/ out of 25 candidates), antineoplastics (9/16), natural plant products (14/17), mTOR related compounds (5/14), NF-kβ inhibitors (3/10), NSAID COX inhibitors (5/7), and estrogen receptor agonists (4/6). Despite these results and the consistent response in our assay to the established MCT1 inhibitor AZD3965, the presence of false negatives within the library cannot be fully ruled out due to the use of a chimeric *R*. *norvegicus* MCT1 protein. In contrast, false positives were primarily antifungals, antineoplastics and NF-kβ inhibitors, many with higher apparent activity than AZD3965, and were easily identifiable through cytotoxicity independent of MCT1 activity. From the positive hits, many molecules were identified that are previously known or related to described MCT inhibitors including several natural products. Apigenin, biochanin A, naringenin (flavonoid), and quercetin (chalcone) are known natural product non-selective MCT inhibitors, with the related compounds 4-hydroxyflavonone and 4-hydroxychalcone showing the highest potency of all the screened natural products [31–33]. Despite this, these compounds exhibit only moderate activity and flavonoid tolerance in humans is not well defined, although they have reported anticancer/antineoplastic activity [34,35]. However, there is still therapeutic potential for these molecular scaffolds, as a similar synthetic derivative of a natural product, a-cyano-4-hydroxycinnamic acid, was modified from a weak to nanomolar inhibitor of both MCT isoforms [14,16]. Of the remaining inhibitors, those with the highest clinical relevance are the NSAID family.

NSAIDs have long been associated with cancer prevention due to the link between inflammation and cancer, but they also show promise in cancer treatment through inhibiting tumor cell proliferation and inducing apoptosis [36]. This activity was traditionally thought to be related to COX inhibition–as prostaglandins simulate cell proliferation and reduce apoptosis, while COX2 affects tumor invasiveness. Despite this, higher NSAID concentrations are generally needed for anti-cancer versus anti-inflammatory effect, indicating that this activity may be independent of COX inhibition [37]. This is further supported from research with NSAID derivatives, such as exisulind the sulfone metabolite of sulindac, which displays no COX inhibition yet retains antitumor activity [38]. Following this observation, early characterizations of COX-independent antineoplastic effects were reported with changes in gene expression and pathway activity (NF-KB, peroxisome proliferator-activated receptors (PPAR), wnt and akt pathways), rather than a direct molecular target [39]. It was later noted that diclofenac causes intracellular accumulation of lactate preceding observed changes in gene expression [40–42]. More recently, this lactate inhibition was directly linked to both MCT1 and MCT4, demonstrating that the COX-independent anticancer mechanism of certain NSAIDs may in part be due to inhibition of MCTs [21].

Indeed, inhibition of MCTs by NSAIDs has been known for over 2 decades, with the inhibition of MCT1 by niflumic acid being the first reported instance [31]. This was later extended to other NSAID family members with ketoprofen, indomethacin, and lexoprofen showing MCT4 inhibition [30]. Here we demonstrate that many NSAIDs inhibit lactate transport, with activity towards MCT1, and potential activity towards MCT4. Although MCT4 was not tested in this study, it is possible that many of the NSAIDs identified in this screen inhibit both isoforms. Specifically, NSAIDs with confirmed activity towards MCT1 have similar structures to known non-selective MCT inhibitors. The known nonselective NSAID inhibitor diclofenac is situated closely to other nonselective natural products in multidimensional scaling, similar to how the other NSAID candidates’ group with the previously identified MCT4 inhibitors (diclofenac, ketoprofen, and indomethacin). We posit that this proximity suggests that compounds may behave similarly in terms of inhibition profile. However, this may be restricted to the monocarboxylic acid containing NSAIDs, which likely act as a pseudo substrate for both transporters [43]. Regardless, we were able to show direct MCT1 interaction for four new NSAID compounds (sulindac, piroxicam, meloxicam, celecoxib) and expand the available known scaffolds of MCT inhibitors.

In addition to identification, we were also able to rank these newly identified and known MCT inhibitors in order of relative potency to glean general structure-activity relationships. Many NSAIDs appear to be weakly or non-interacting compounds with aspirin, rofecoxib, mefenamic acid, nabumetone (110 μM IC_50_), ibuprofen (69), naproxen (46), and fenoprofen (42) belonging to this group. Monocyclic NSAIDs appear to generally exhibit negligible activity as aspirin, ibuprofen, and naproxen do not inhibit MCT1. This is in line with previous research that supports that these commonly used NSAIDs are not MCT inhibitors but may rather be transporter substrates [36,44,45]. Other weak inhibitors, sulindac (46 μM IC_50_) and ketoprofen (37) are still promising as they do not exhibit off-target toxicity at the dosages used in this study, unlike those outlined above. Finally, we were able to identify five NSAIDs with moderate activity towards MCT1—piroxicam (4.4 μM IC_50_), diclofenac (7.4), meloxicam (17), indomethacin (27), celecoxib (35). Of these compounds, tricyclic NSAIDs appear to be the most effective structural variants in terms of MCT1 inhibition, with meloxicam and piroxicam forming a structurally related duo with high activity. Interestingly, these compounds display higher activity than the other tested tricyclics (celecoxib, indomethacin, and sulindac), despite not containing a carboxylic acid moiety. It is likely that either these compounds do not act in a competitive manner, or the conserved sulfone moiety is able to provide a suitable carboxylic acid substitution. Thus, many structurally divergent NSAIDs exhibit MCT1 inhibition, and we provide evidence for the first time that sulfone-containing NSAIDs display activity towards MCT1, at concentrations relevant to their anticancer and anti-inflammatory effects.

Although promising, NSAID treatment is associated with significant gastro-toxicity related to COX1 inhibition, and all NSAIDs have cardiovascular risks including myocardial infarction and stroke [46,47]. However, NSAIDs vary in their COX1/2 inhibitory profile potency, with COX-2 selective inhibitors showing promise to treat cancer while addressing gastric side-effects [48]. For example, nontoxic dosages of the COX2-selective NSAID diclofenac are effective in reducing glioma growth and lactate concentrations in vivo [41,49]. Of the available inhibitors diclofenac appears to have the greatest COX2 selectivity; however, another study showed meloxicam and celecoxib to be the most selective with similar ratios between diclofenac and piroxicam [50–52]. Interestingly, COX2 selectivity may relate to MCT inhibition as four out of the five strongest inhibitors identified in our study (Piroxicam, diclofenac, meloxicam, and celecoxib) also display strong preference for COX2. Of these compounds, piroxicam is one of the most interesting new MCT inhibitors due to its high bioavailability following dosage relative to other NSAIDs. While 150–200 mg daily of diclofenac produces a blood-plasma concentration of 6.1 μM, only 20 mg/day of piroxicam produces a concentration of 16–20 μM, which is well within the range necessary for MCT inhibition [50,53]. Furthermore, despite NSAIDS generally needing a higher dosage for anticancer activity, this may not be true for all NSAIDs as piroxicam displays in vitro anticancer activity at concentrations relevant to MCT1 inhibition (25–30 μM) [47]. Finally, despite the noted side-effects NSAIDs are generally well tolerated and could replace syrosingopine for MCT inhibition in combined treatment with metformin [10,11]. Overall, NSAIDs represent a diverse well-tolerated molecular class with readily available molecules (7 FDA approved compounds) exhibiting MCT1 inhibition.

## Methods

### Drugs and candidate molecules

The drug library used in this study for the initial screen and rescreen was provided by the Small Molecule Screening Center at Princeton University, through a collaboration with Dr. Hahn Kim. Library compounds (10 mM concentration in dimethyl sulfoxide (DMSO)), were dispensed at 0.05 μl into a 384 well plate using a Labcyte ECHO 550 acoustic dispenser to result in a final concentration of 10 μM (assays were performed in a 50 μl volume). Follow-up analysis (IC_50_ determinations) on library compounds was performed using compounds purchased from outside sources. AZD3965, celecoxib, diclofenac diethylamine, fenoprofen, ketoprofen, mefenamic acid, meloxicam, nabumetone, nordihydroguaiaretic acid, rofecoxib, and sulindac were purchased from Ambeed. 4-hydroxychalcone, ibuprofen, and piroxicam were purchased from AA Blocks. Aspirin, (+)-naproxen, and syrosingopine were purchased from Cayman Chemical Company. Finally, indomethacin was purchased from Santa Cruz Biotechnology and 4-hydroxyflavonone from MedChem Express. For determination of IC_50_ values, chemical stocks were prepared at a 1 mM concentration in DMSO and diluted 1:10 with DMSO to achieve each stock concentration. Stocks were then diluted 100-fold into media containing 0.01 OD600 inoculation of yeast, to achieve the final concentrations listed. Where compounds were added manually (for example 10 μM AZD3965 addition) stock concentrations were diluted by a factor of 100-fold regardless of scale, resulting in a final concentration of 1% v/v DMSO which is well tolerated by yeast [54].

### Plasmids

All plasmids used in the current study were derivatives of the pJLA series of vectors, using established pairs of promoters and terminators, with the only modification being the replacement of the high-copy 2μ plasmid origin with the low-copy CEN/ARS origin where specified [55,56]. The *R*. *norvegicus* MCT1 sequence (Uniprot: P53987) was codon-optimized for expression in *S*. *cerevisiae* and synthesized by Twist Biosciences. All cloning was performed with isothermal assembly, following either enzymatic digestion or PCR using Takara CloneAmp HiFi polymerase, according to the Gibson method [57]. Introduction of the F360C mutation was performed using primers 5-TTCGCCT**G**CGGCTGGCTAAGTTCAGTATTATTCGAG-3 and 5’-CAGCCG**C**AGGCGAATCCAAAGACGCCTG-3, where the bolded base reflects the coding mutation introduced. Quickchange PCR was performed by amplifying the wildtype sequence with 25 cycles of PCR, digestion of the non-desired parent product with DPNI, then transformation and verification of the introduced mutation with sanger sequencing. All vectors were sequenced with Sanger sequencing (GENEWIZ) to verify integrity of the coding regions. Plasmids were constructed using chemically competent DH5a strains grown at 37°C in LB media with 100 ug/ml ampicillin [58].

C-terminal tags were added downstream the coding region of MCT1 immediately preceded by a 15 amino acid long flexible linker (GASAGGSAGGSAGSGG), using the previously published plasma membrane association module from *GAP1* [23]. Alternatively, the c-terminal cytosolic segments of *JEN1*, *HXT6*, and *PDR5* were identified using a hydropathy plot performed in ProtScale (Expasy) with the Kyte and Doolittle scale [59]. The c-terminal final hydrophilic domain of each protein was added to MCT1 downstream of the described linker. For protein visualization green fluorescent protein (GFP) was added downstream of the MCT1 coding region with the same flexible linker as mentioned, with c-terminal tags added immediately downstream of GFP. All c-terminal tags were amplified from CEN.PK2-1c genomic DNA. N-terminal tags were added as direct fusions immediately upstream the MCT1 coding region, with removal of the redundant start codon from MCT1. The invertase *SUC2* tag was amplified from the yeast genome [60]. The remaining tags AFROPT and SMD154b were synthesized by Twist Biosciences [24,25]. MCT1 chimeras were constructed using the n-terminal 95–133 region of *JEN1* and the c-terminal 555–583 region, previously reported to support plasma membrane localization [26].

### Yeast medium and strain construction

Yeast was propagated, transformed, and assayed in synthetic complete media. Synthetic media was composed of 1.5 g Difco yeast nitrogen base without amino acids and ammonium sulfate, 5 g ammonium sulfate, 200 μM inositol, and 2 g amino acid powder mix per liter. For all studies using MCT1, uracil was excluded to maintain plasmid selection. The carbon source for most experiments was 2% glucose; however, for growth in alternative carbon sources lactate (0.5% v/v) or pyruvate (40 mM) was used instead with media pH adjustment to 5.0. For media containing mevalonate, mevalonolactone (Acros Organics) was saponified to mevalonate, with 1.5:1 mol KOH:mevalonolactone suspended in MilliQ water with incubation at 37°C for 2 hours. This solution was then added to the media, for a final concentration of 10 mM mevalonate, and the pH was adjusted to pH 5.0 using 12N HCL.

Yeast was transformed following the established Lithium acetate procedure, with the relevant plasmids listed in S7 Table and the resulting strains listed in S8 Table [61]. Most yeast strains used in the current paper were constructed previously (SAWy119, SAWy518, SAWy524; Wegner SA, Jiang V, Cortez JD, Avalos JL. [In preparation]). In brief, for the optogenetic strains, CEN.PK2-1C was modified to express asLOV2 and an optoINVRT1 circuit, with deletion of gal4/gal80 (SAWy119) [55]. The protein paralog HMG2 was deleted by homologous recombination with an antibiotic resistance marker (SAWy518), and the HMG1 promoter was replaced by pC120 to allow blue-light mediated expression, resulting in the optoMEV strain (SAWy524). For growth in lactate or pyruvate as a sole carbon source by4741 and an equivalent strain with a *JEN1* knockout was obtained from the nonessential gene deletion library [62].

### Microscopy

The yeast strain for colocalization to the ER, containing an ER-retained HDEL-mCherry, was previously [63]. Strains were transformed with the GFP-tagged MCT1 variants and stained with MemBrite 640/660 (Biotium) a membrane dye to measure correlation with the plasma membrane. Microscopy was performed following our previously established methods [63]. Images were acquired using a Nikon W1 SoRa microscope with a 63X oil objective and 4X magnification. For image analysis, cells were first segmented using ImageJ to define the region of interest. Colocalization metrics were performed using the JaCoP plugin to determine both the Person’s R correlation between the MCT-GFP channel and the plasma membrane as well as the Manders M1 and M2 coefficients of signal overlap [64].

### Optogenetic growth and high throughput growth

Low throughput conditions to determine growth and growth rate were performed with biologically independent transformants grown overnight (~16 hours) in full blue light (40–60 μmol m^-2^ sec^-1^ as measured by an Apogee quantum meter (MQ-510)), delivered by a blue light-emitting diode (LED) panel (HQRP 12-inch blue LED 14W). Overnights were then diluted normalized to ~ 1.0 OD prior to back dilution for growth. Growth was determined from a 0.01 starting OD in 1 ml synthetic media in a 24-well plate, with incubation conditions being 30°C, 200 RPM, and either blue light or covered with Aluminum foil. Optical density was measured periodically using a Tecan Infinite F Plex reader with appropriate filter set (600 (10)), with a blank well in each plate to assess contamination. All OD600 data is presented as raw Tecan measurements. Following saturation, the growth rate was determined using the early exponential log-linear portion of the growth curve, with the slope being the reported specific growth rate (μ).

High throughput experiments were performed using the same medium, outgrowth, and starting dilutions as the low throughput method but in 96 or 384-well format. Different volume (25, 50, 100 μl) and aeration conditions were also tested for growth at 30°C: either 200 RPM shaking conditions versus unshaken conditions, with or without a plate seal to allow higher gas exchange. Growth was measured at 24 hours. Z-factor determination was performed using the 50 μl 384-well format without shaking. The growth assay was performed independently three times, starting from unique inoculations, with fresh media prepared for each assay. Assays were then incubated first a 24-hour period at 30°C covered with foil, and a subsequent 24-hour incubation under blue light (20–40 μmol m^-2^ sec^-1^). Optical density was measured after each 24-hour period with plates resuspended by rapid mixing at 2000 RPM for 15 seconds.

The IC_50_ for each candidate compound identified from the library was also performed using the 384-well assay. Compounds were procured and diluted as described above. The compounds were then manually added to the 384-well plate (0.5 μl/well), following which 50 μl of synthetic media containing 10 mM mevalonate was added. Prior to addition of media to the plate either the optoMEV (SAWy524) or the light-insensitive parent strain (SAWy518) was inoculated into the media at a starting dilution of 0.01 OD600.

### Library screening and rescreening

The drug library was supplied in 384-well format with 0.05 μl (10 μM, in DMSO) of each test compound added to the plate. Library plates were stored at 4°C until use and were used within 48 hours of receipt. Immediately prior to use 0.5 μl of either DMSO (negative control) or 10 μM AZD3965 (positive control) were each added to 4 wells of every plate. The high-throughput assay format was the same as described above (384-well, 10 mM mevalonate, 0.01 starting SAWy524 inoculation). Plates were then grown for 24 hours in non-permissive dark conditions, then transferred to permissive conditions for an additional 24 hours. The light intensity in the permissive phase varied from 14–66 with an average intensity of 40 μmol m^-2^ sec^-1^. At the end of each phase plates were mixed briefly at 2000 RPM for 15 seconds and OD600 was measured. The Z-score for each compound was calculated relative to all test compounds within a 384-well plate and was first normalized by dividing the growth in the non-permissive phase by growth in the permissive phase. To calculate the relative potency of each compound a “percent AZD3965” score was calculated by using DMSO as the upper bound of growth (0% AZD3965) and AZD3965 as the lower bound (100% AZD3965).

The secondary screen was performed similarly to the library screen. Library compounds were re-acquired from the Small Molecule Screening Center stocks in the same volume and concentrations as described above. However, instead of the optogenetic strain, the parent strain (SAWy518) was used and subject to the same growth conditions as the primary screen: 0.01 starting OD, 50 μl 384-well format, and the same media as the primary screen. However, the media was supplemented with uracil to account for the auxotrophic requirement of the parent strain (1:100 dilution of a sterilized 20 mM stock). Plates were incubated at 30°C covered with foil with measurements at 18, 24, and 48 hours.

### Molecular fingerprinting, hierarchical clustering, and multidimensional scaling

The initial molecular grouping was performed manually using compound activity annotations provided by Dr. Hahn Kim. Compounds were grouped based on recurring families (such as natural products) or targets of interest, with reported grouping based on the most frequently observed classes. All other compounds which did not have an apparent grouping were assigned to the “other” category. Structural comparisons were performed using the ChemMine web portal [65]. Compounds which were represented more than once within the library were collapsed into a single identifier (86 total, 76 unique), using the lowest observed Z-score for any repeats. Compounds were first separated into single linkage hierarchical clusters based on compound similarity defined using atom pair descriptors and Tanimoto coefficient. Families were defined manually using the observed hierarchical tree, and appropriate branchpoints. Families were visualized using 2D multidimensional scaling (0.4 similarity cutoff).

### Data analysis

Single group comparisons were performed in Microsoft Excel using a two-tailed t-test. For multiple comparisons, a one-way ANOVA was performed with Tukey post-hoc correction. Pathway figures were created using Biorender. Graphs were generated in Excel, R using the ggplot2 package, or in GraphPad Prism for IC_50_ determination. IC_50_ was determined in GraphPad using a nonlinear regression. Protein structures were obtained from AlphaFold, while structures of molecules were obtained from ChemMine. Finally, structural comparisons of JEN1 and MCT1 were performed in UCSF Chimera.

## Supporting information

S1 Fig MCT1 expression in *Saccharomyces cerevisiae* primarily localizes to the endoplasmic reticulum (ER). **A)** Localization of the unmodified MCT1 construct from *Rattus norvegicus* visualized with a C-terminal GFP tag. Compartmentalization is assessed by signal overlap with either HDEL-mCherry **(B)** or Membrite 640 dye **(C)** to analyze colocalization with the ER or plasma membrane, respectively. **D)** The overlap of all channels shows accumulation of MCT1-GFP within the ER, depicted in yellow.
(TIF)

S2 Fig GAP1c facilitates ER-exit of the MCT1 construct, while other c-terminal tags remain localized to cellular substructures. **A)** The fluorescent signal of MCT1-GFP tagged with c-terminal regions of specified endogenous transporters, with correlation and overlap with the plasma membrane shown in **(B)**. Growth curves were conducted to assess functional monocarboxylate transport with either pyruvate **(C)** or lactate **(D)**. The growth of these strains is impeded by the knockout of the *JEN1* transporter, which normally mediates monocarboxylate import and supports growth (*Δjen1*). *p < 0.05, *** p < 0.001, **** p < 0.0001.
(TIF)

S3 Fig The N-terminal SUC2 tag enables MCT1 protein function in *S*. *cerevisiae*. **A)** The fluorescent signal of MCT1-GFP tagged with the C-terminal region of *JEN1* and/or the N-terminal SUC2 secretion tag. The correlation and overlap with the plasma membrane are shown in **(B)**. Growth curves were conducted to assess functional monocarboxylate transport with either pyruvate **(C)** or lactate **(D)**. The growth of these strains is impeded by the knockout of the *JEN1* transporter, which normally mediates monocarboxylate import and supports growth (*Δjen1*). *** p < 0.001.
(TIF)

S4 Fig Domain fusions from *JEN1* yield functional MCT1 chimeras. **A)** The complete predicted alpha-fold structure is depicted for both proteins, with the cytosolic termini indicated in blue and red for the N-terminus or C-terminus, respectively. Different MCT1 substitutions are highlighted with colors corresponding to the region to be replaced. **B)** Growth of the optoMEV strain with various JEN1-MCT1 chimeras. Growth was assessed in non-permissive conditions in media supplemented with 10 mM mevalonate.
(TIF)

S5 Fig Growth of the optimal MCT1 fusion in lactate and sensitivity to AZD3965 administration. The optimal construct (JEN1n-MCT1(F360C)) was expressed in the monocarboxylate transport deficient background (*Δjen1*), with 0.5% w/v lactate supplied as the sole carbon source. As a control, an equivalent wild-type construct (all above constructs not specified contain the F360C mutation) was also created to demonstrate that the standard AZD3965 inhibitor would be effective on both the mutant and wildtype forms of MCT1. AZD3965 was added to the media at a concentration of 10 μM.
(TIF)

S6 Fig High throughput assay optimization in either 96-well **(A)** or 384-well **(B)** format. The optoMEV strain containing either the optimal MCT1 chimera (SAWy741) or an empty vector (SAWy710) was treated either with the specific inhibitor AZD3965 (10 μM) or an equivalent volume of vehicle (DMSO). The assay conditions were varied to study different assay volumes (25, 50, 100 μl) and aeration conditions. Non-shaking cells were allowed to settle throughout growth, while shaking cells were grown at 200 RPM. Following 24 hours of incubation at 30°C, OD600 was measured.
(TIF)

S7 Fig Assay robustness for three independent MCT1 inhibitor assays. The optimal incubation condition (384-well plate, 50 μl assay volume, without shaking) was used to determine the effect size between MCT1 inhibition (Orange, AZD3965 10 μM) versus no inhibition (Blue, DMSO). The individual growth values for 10 replicates are shown with gray highlighting representing 3 standard deviations above and below the mean for each treatment. This data was used to calculate the average Z-factor score from the individual results seen here.
(TIF)

S8 Fig Additional controls included with the library screening. In addition to the positive (AZD3965; AZD) and negative (DMSO) wells for each drug library plate, additional controls were included in a separate plate to ensure the library exhibited a similar level of assay robustness. **A-B)** The experiment was performed as in Fig 3D, with two replicates subjected to different incubation conditions. While replicate 1 was transferred to permissive conditions following 24 hours of incubation **(A)**, replicate 2 remained at non-permissive conditions throughout the 48-hour period **(B)**.
(TIF)

S9 Fig General cytotoxicity of all the putative MCT1 inhibitors identified in the primary screen. The 154 candidate molecules (Library) were rescreened for growth inhibition using a yeast strain that does not require light or MCT1 activity (SAWy518). The candidates were compared for growth inhibition relative to the known specific inhibitor AZD3965 (10 μM), which should not affect the growth of this strain, and non-treated wells (Empty). Following incubation in dark conditions, OD600 was measured after 18 hours **(A)**, 24 hours **(B)**, and 42 hours **(C)**. The dashed line reflects the lower bounds for being within 10% of the average OD600 of untreated wells. **D)** shows the correlation between 18-hour versus 24-hour (filled dots) or 42-hour (open dots) OD600 measurements.
(TIF)

S10 Fig Hierarchical clustering of the 76 identified MCT1 inhibitors. The table shows the molecular similarity based on the Tanimoto method (see methods) with self-comparisons shown in blue.
(TIF)

S11 Fig Hierarchical clustering of all tested NSAID compounds, defined by annotated COX inhibition. The table shows the molecular similarity based on the Tanimoto method (see methods) with self-comparisons shown in blue.
(TIF)

S1 Table Growth rate of optoMEV and parent strains. (DOCX)

S2 Table Growth rates of 2μ unmodified MCT1 constructs. (DOCX)

S3 Table SUC2n-MCT1-GAP1c growth rate in lactate. (DOCX)

S4 Table Growth rates of tagged CEN/ARS constructs. (DOCX)

S5 Table Growth rates of chimeric CEN/ARS constructs. (DOCX)

S6 Table Growth rates withAZD3965 treatment. (DOCX)

S7 Table Plasmids used in this study. (DOCX)

S8 Table Yeast strains used in this study. (DOCX)

S1 File This date file contains the information and screening data of all compounds tested in the current study. The final list contains all compounds which pass both the Z-score criterion (Z < -3) in the initial screen, as well as the growth criterion (> 90%) in the secondary screen.
(XLSX)

S1 Data This data file contains the data used for the generation of all figures presented in the current study. (XLSX)
