# Supplementary figures and images for: Optogenetic screening of MCT1 activity implicates a cluster of non-steroidal anti-inflammatory drugs (NSAIDs) as inhibitors of lactate transport
Source: PLoS One. 2024 Dec 12;19(12):e0312492. doi: 10.1371/journal.pone.0312492 (PMC11637378; doi:10.1371/journal.pone.0312492)

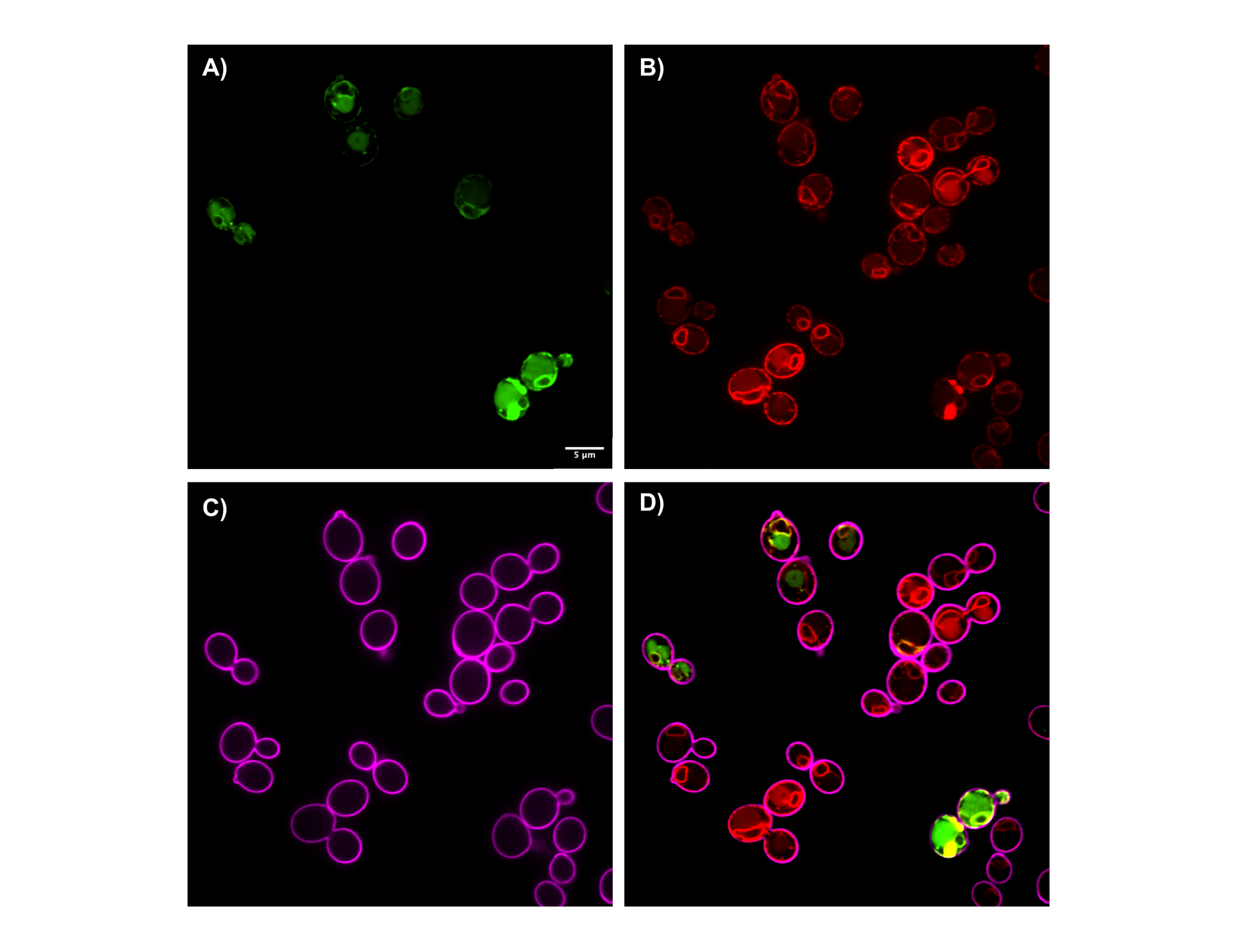

Supplement: S1 Fig — A) Localization of the unmodified MCT1 construct from Rattus norvegicus visualized with a C-terminal GFP tag. Compartmentalization is assessed by signal overlap with either HDEL-mCherry (B) or Membrite 640 dye (C) to analyze colocalization with the ER or plasma membrane, respectively. D) The overlap of all channels shows accumulation of MCT1-GFP within the ER, depicted in yellow. (TIF) [file pone.0312492.s001.tif]

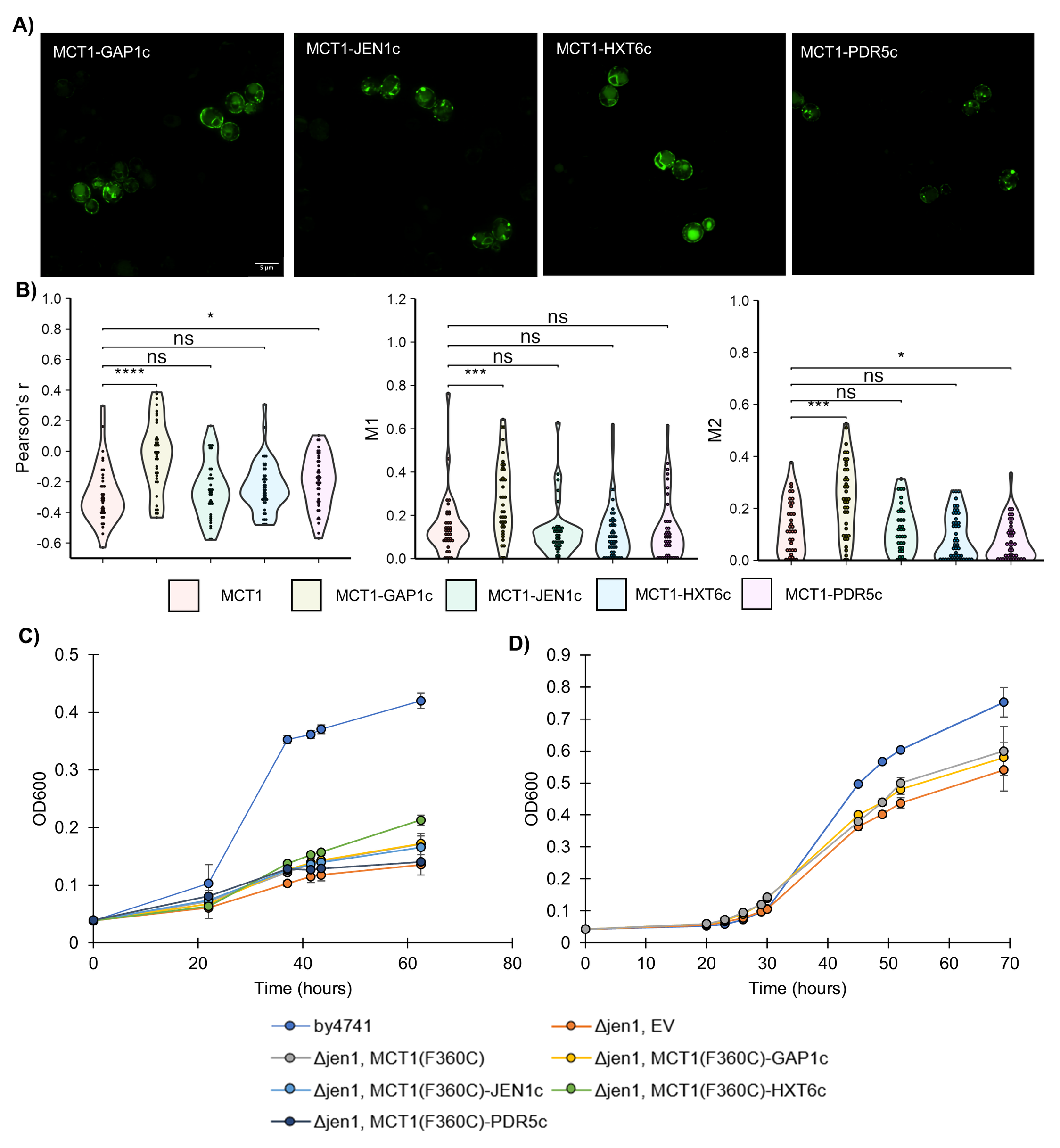

Supplement: S2 Fig — A) The fluorescent signal of MCT1-GFP tagged with c-terminal regions of specified endogenous transporters, with correlation and overlap with the plasma membrane shown in (B). Growth curves were conducted to assess functional monocarboxylate transport with either pyruvate (C) or lactate (D). The growth of these strains is impeded by the knockout of the JEN1 transporter, which normally mediates monocarboxylate import and supports growth (Δjen1). *p < 0.05, *** p < 0.001, **** p < 0.0001. (TIF) [file pone.0312492.s002.tif]

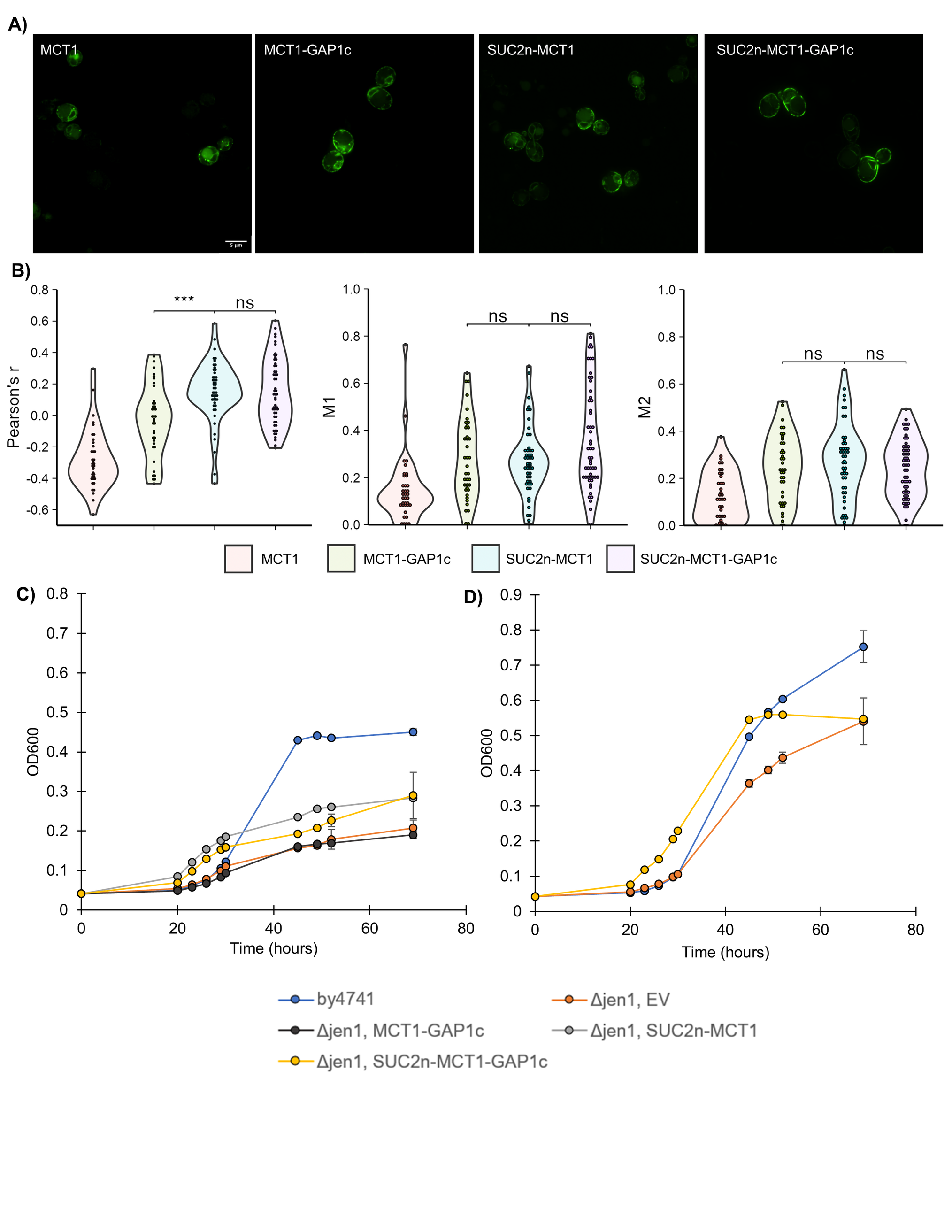

Supplement: S3 Fig — A) The fluorescent signal of MCT1-GFP tagged with the C-terminal region of JEN1 and/or the N-terminal SUC2 secretion tag. The correlation and overlap with the plasma membrane are shown in (B). Growth curves were conducted to assess functional monocarboxylate transport with either pyruvate (C) or lactate (D). The growth of these strains is impeded by the knockout of the JEN1 transporter, which normally mediates monocarboxylate import and supports growth (Δjen1). *** p < 0.001. (TIF) [file pone.0312492.s003.tif]

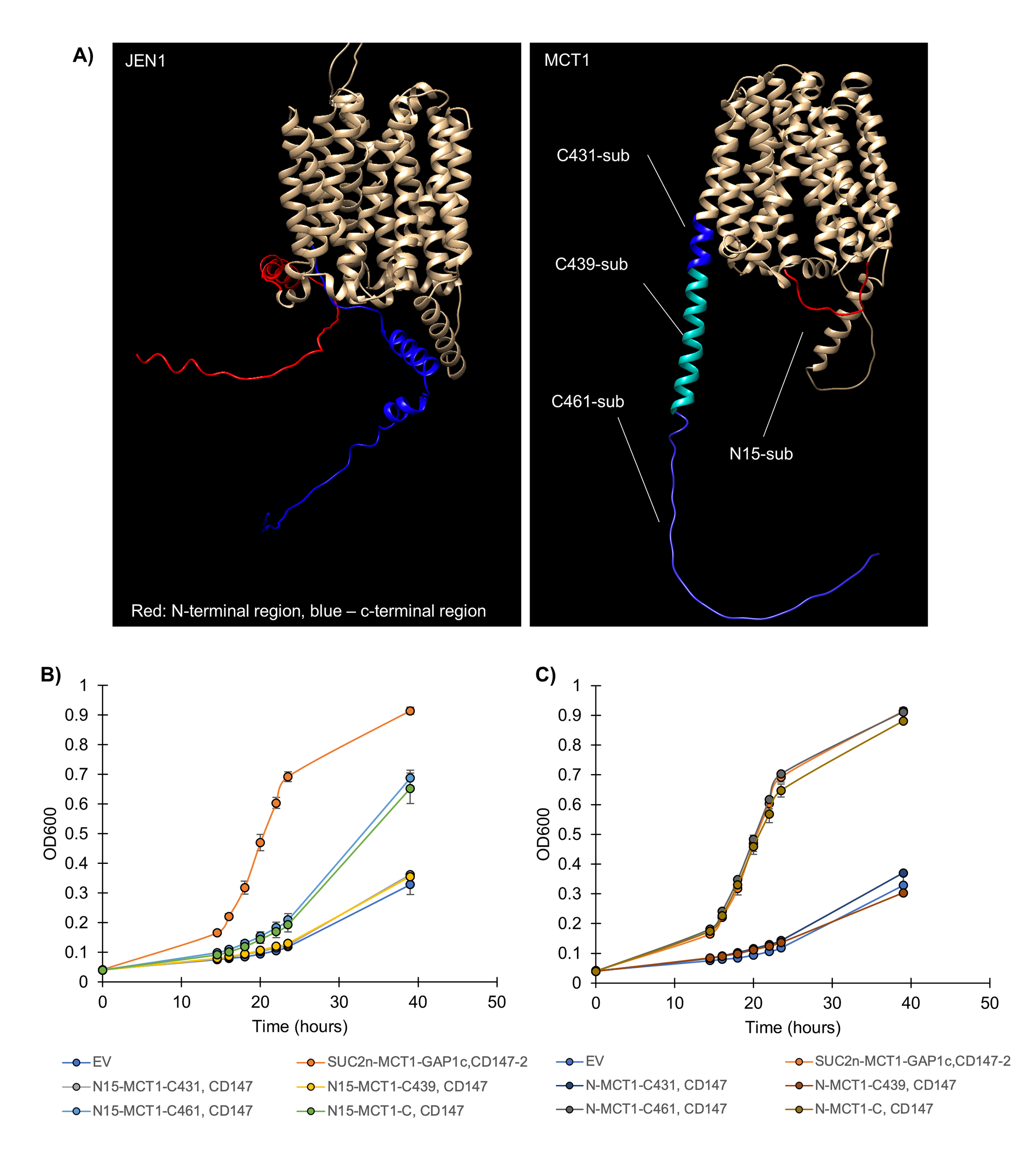

Supplement: S4 Fig — A) The complete predicted alpha-fold structure is depicted for both proteins, with the cytosolic termini indicated in blue and red for the N-terminus or C-terminus, respectively. Different MCT1 substitutions are highlighted with colors corresponding to the region to be replaced. B) Growth of the optoMEV strain with various JEN1-MCT1 chimeras. Growth was assessed in non-permissive conditions in media supplemented with 10 mM mevalonate. (TIF) [file pone.0312492.s004.tif]

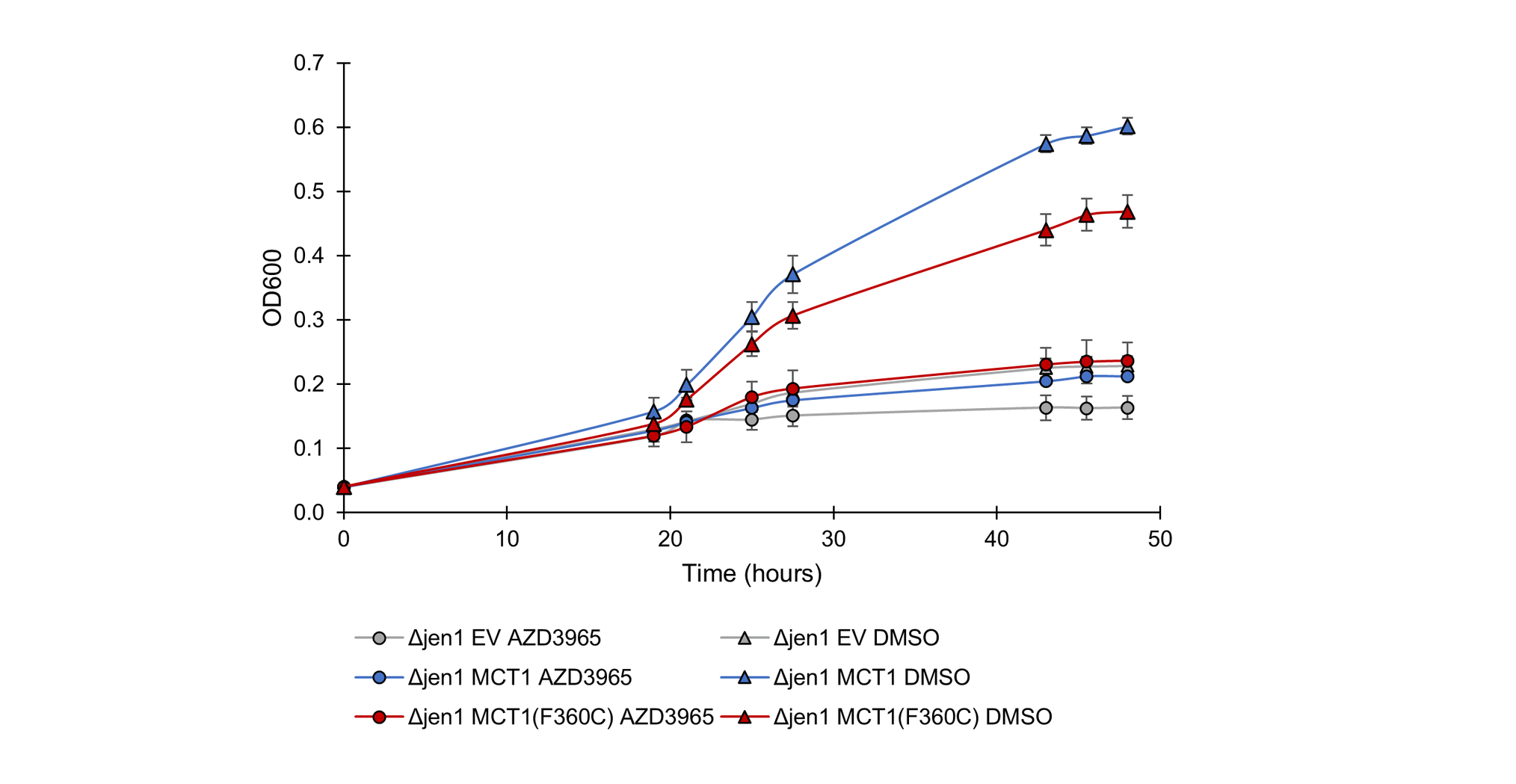

Supplement: S5 Fig — The optimal construct (JEN1n-MCT1(F360C)) was expressed in the monocarboxylate transport deficient background (Δjen1), with 0.5% w/v lactate supplied as the sole carbon source. As a control, an equivalent wild-type construct (all above constructs not specified contain the F360C mutation) was also created to demonstrate that the standard AZD3965 inhibitor would be effective on both the mutant and wildtype forms of MCT1. AZD3965 was added to the media at a concentration of 10 μM. (TIF) [file pone.0312492.s005.tif]

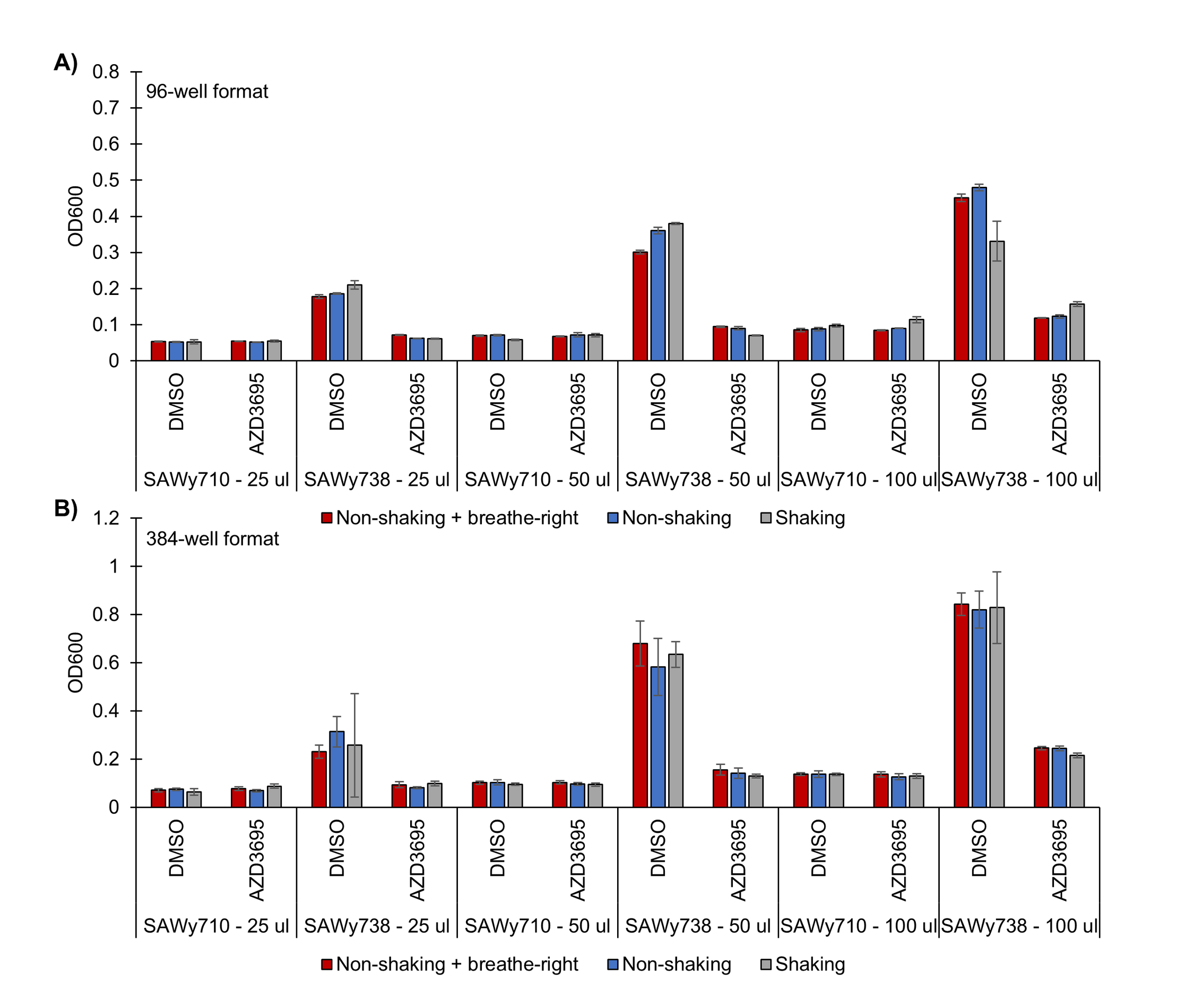

Supplement: S6 Fig — High throughput assay optimization in either 96-well (A) or 384-well (B) format. The optoMEV strain containing either the optimal MCT1 chimera (SAWy741) or an empty vector (SAWy710) was treated either with the specific inhibitor AZD3965 (10 μM) or an equivalent volume of vehicle (DMSO). The assay conditions were varied to study different assay volumes (25, 50, 100 μl) and aeration conditions. Non-shaking cells were allowed to settle throughout growth, while shaking cells were grown at 200 RPM. Following 24 hours of incubation at 30°C, OD600 was measured. (TIF) [file pone.0312492.s006.tif]

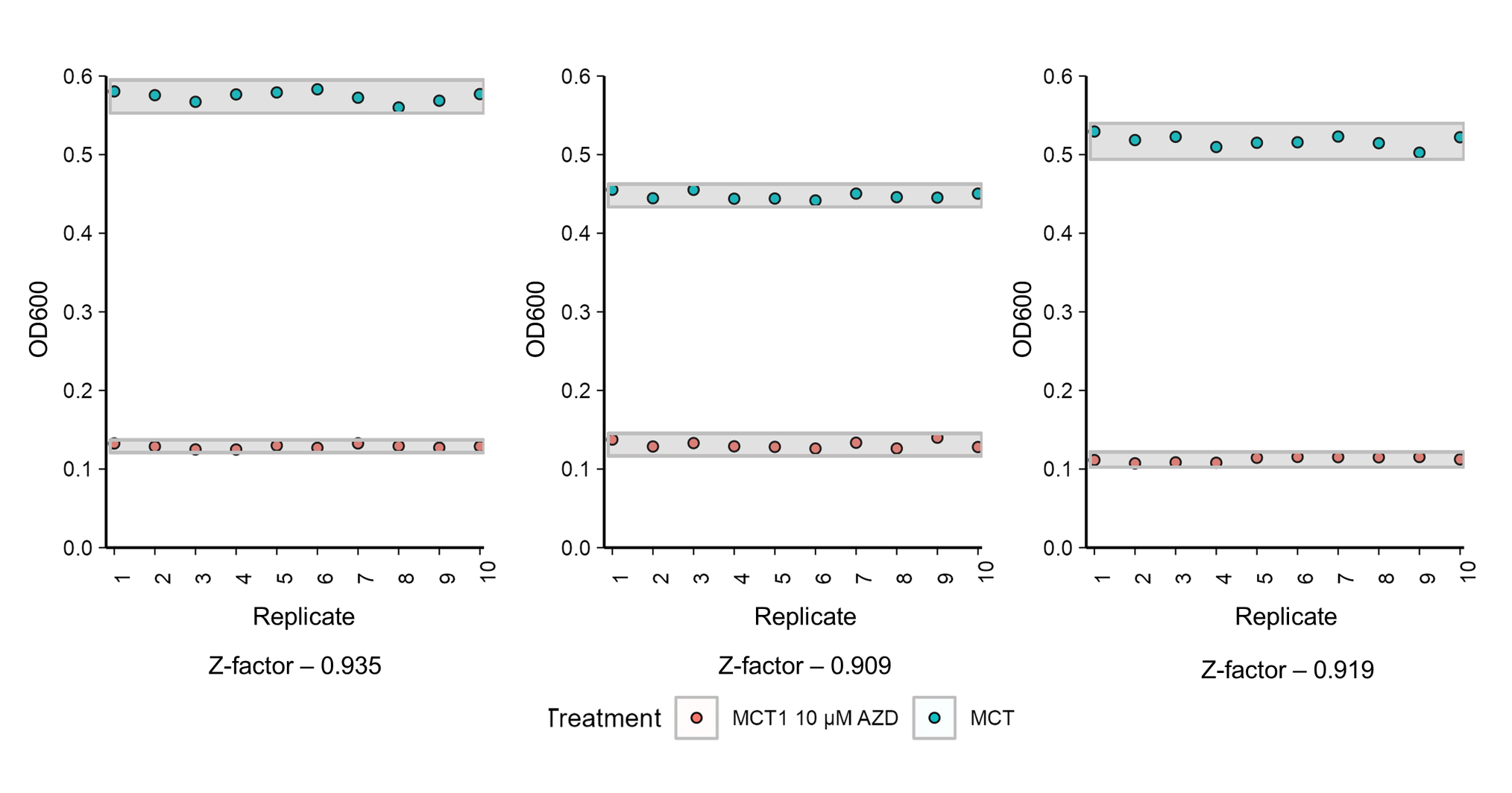

Supplement: S7 Fig — The optimal incubation condition (384-well plate, 50 μl assay volume, without shaking) was used to determine the effect size between MCT1 inhibition (Orange, AZD3965 10 μM) versus no inhibition (Blue, DMSO). The individual growth values for 10 replicates are shown with gray highlighting representing 3 standard deviations above and below the mean for each treatment. This data was used to calculate the average Z-factor score from the individual results seen here. (TIF) [file pone.0312492.s007.tif]

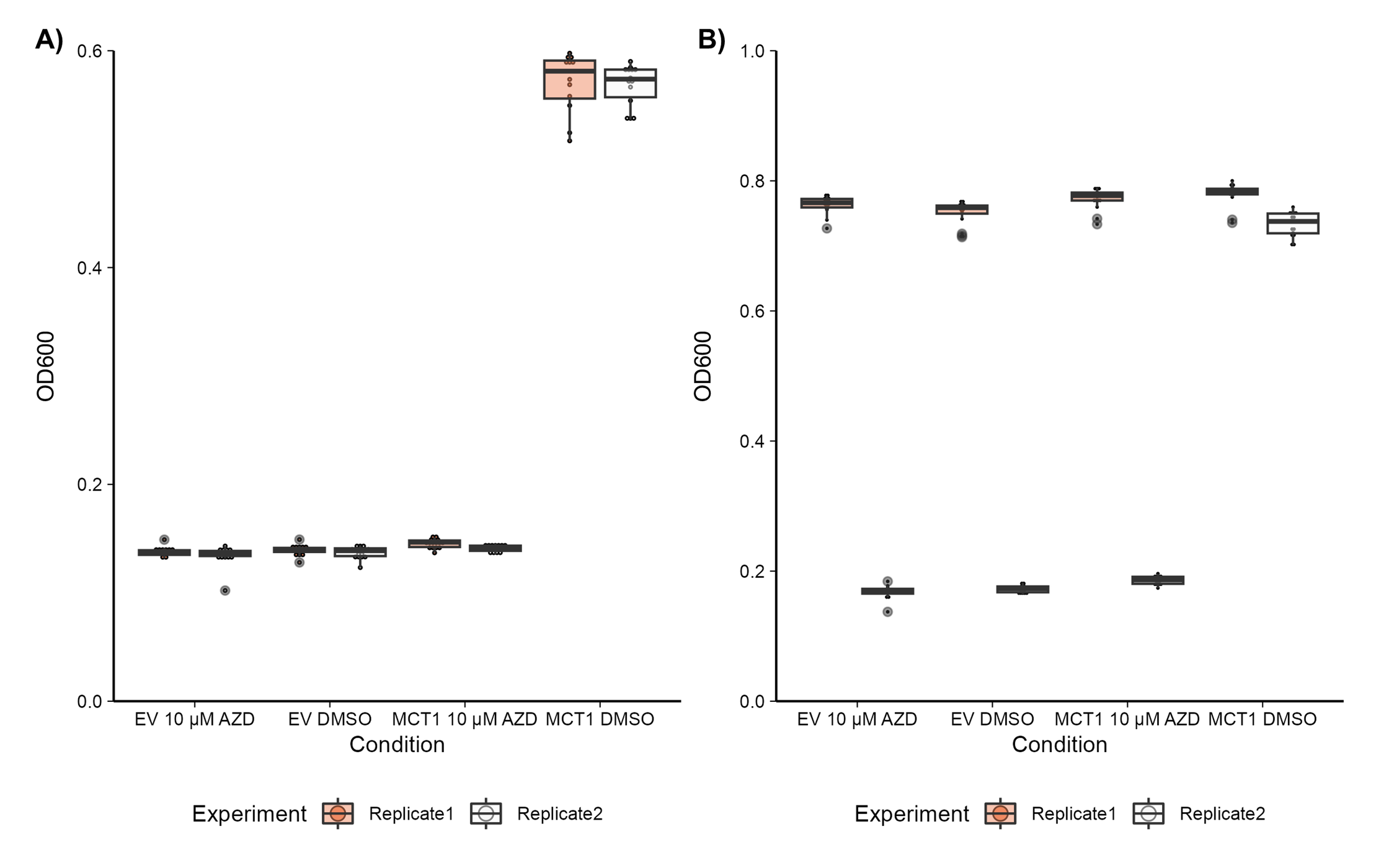

Supplement: S8 Fig — In addition to the positive (AZD3965; AZD) and negative (DMSO) wells for each drug library plate, additional controls were included in a separate plate to ensure the library exhibited a similar level of assay robustness. A-B) The experiment was performed as in Fig 3D, with two replicates subjected to different incubation conditions. While replicate 1 was transferred to permissive conditions following 24 hours of incubation (A), replicate 2 remained at non-permissive conditions throughout the 48-hour period (B). (TIF) [file pone.0312492.s008.tif]

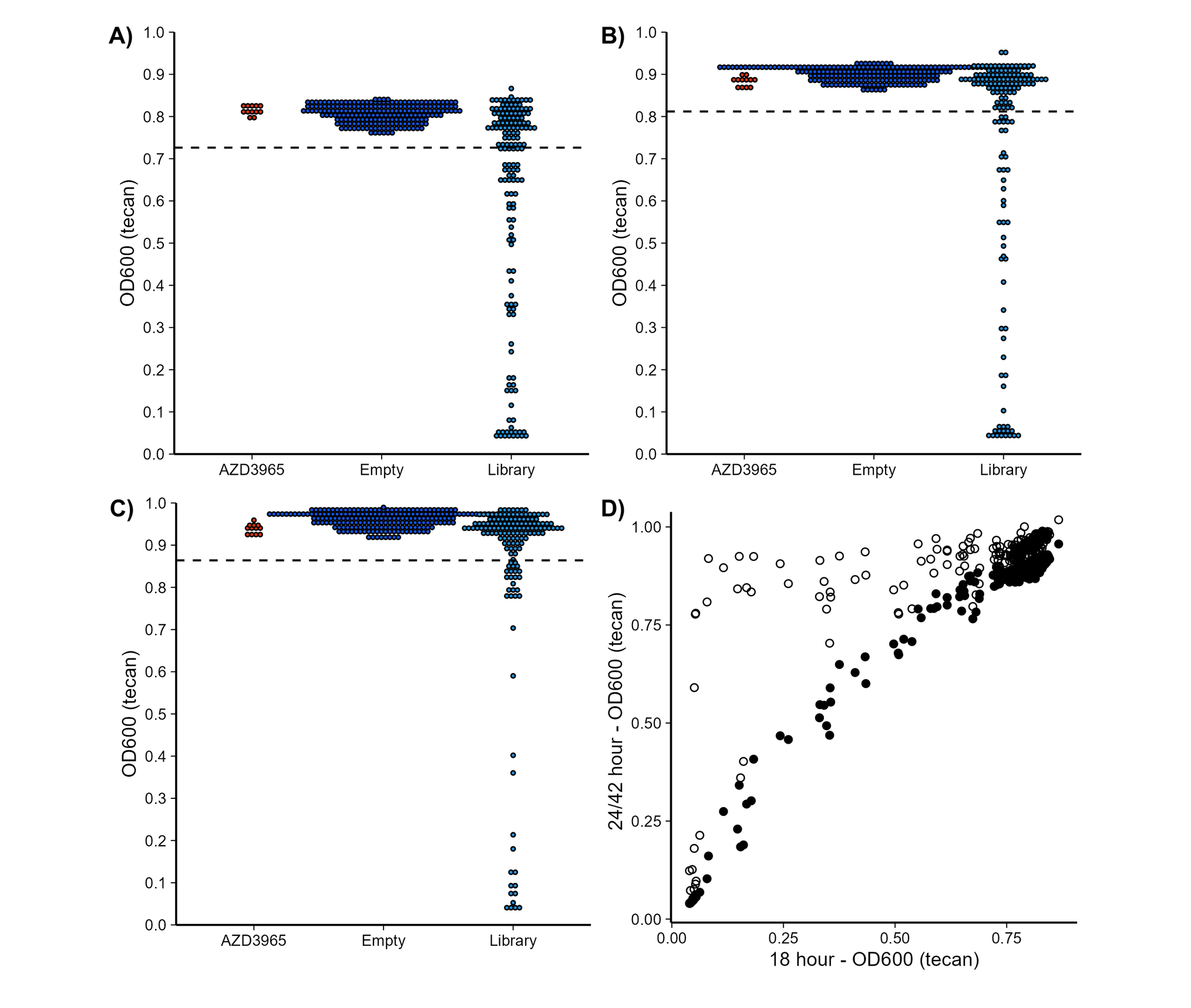

Supplement: S9 Fig — The 154 candidate molecules (Library) were rescreened for growth inhibition using a yeast strain that does not require light or MCT1 activity (SAWy518). The candidates were compared for growth inhibition relative to the known specific inhibitor AZD3965 (10 μM), which should not affect the growth of this strain, and non-treated wells (Empty). Following incubation in dark conditions, OD600 was measured after 18 hours (A), 24 hours (B), and 42 hours (C). The dashed line reflects the lower bounds for being within 10% of the average OD600 of untreated wells. D) shows the correlation between 18-hour versus 24-hour (filled dots) or 42-hour (open dots) OD600 measurements. (TIF) [file pone.0312492.s009.tif]

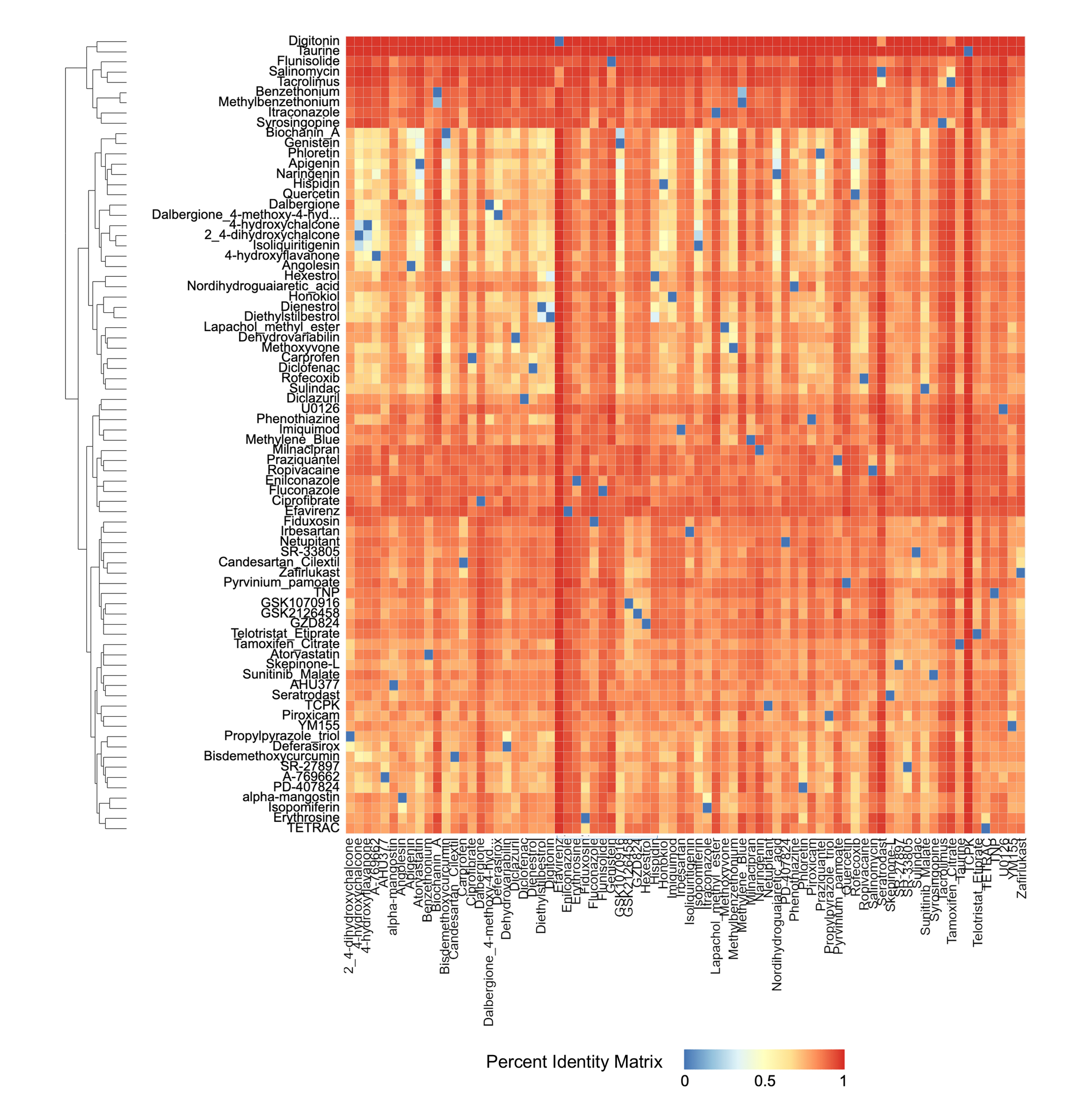

Supplement: S10 Fig — The table shows the molecular similarity based on the Tanimoto method (see methods) with self-comparisons shown in blue. (TIF) [file pone.0312492.s010.tif]

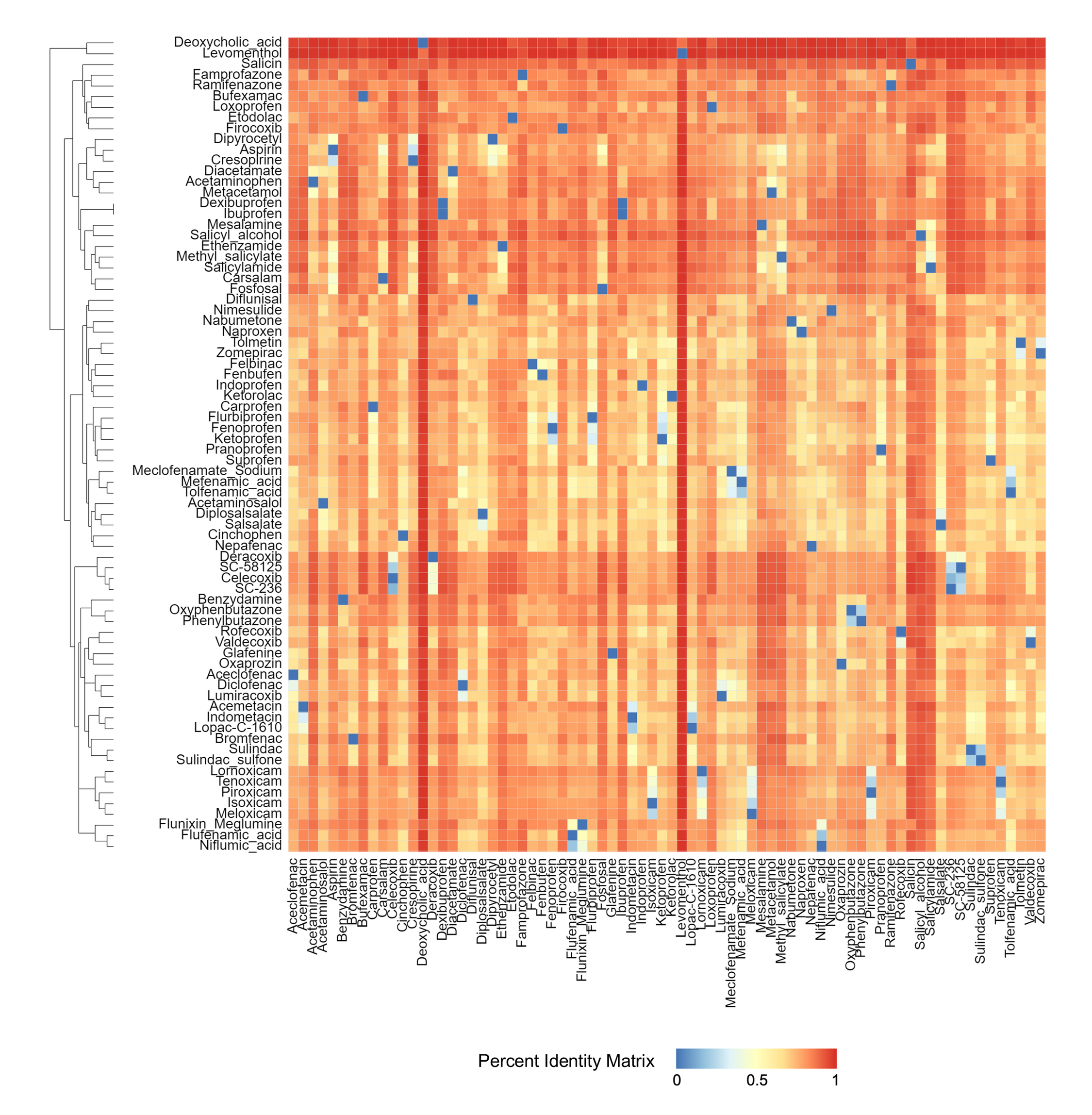

Supplement: S11 Fig — The table shows the molecular similarity based on the Tanimoto method (see methods) with self-comparisons shown in blue. (TIF) [file pone.0312492.s011.tif]
