# Supplementary material for: Optogenetic screening of MCT1 activity implicates a cluster of non-steroidal anti-inflammatory drugs (NSAIDs) as inhibitors of lactate transport
Source: PLoS One. 2024 Dec 12;19(12):e0312492. doi: 10.1371/journal.pone.0312492 (PMC11637378; doi:10.1371/journal.pone.0312492)
Supplement: S1 Table — (DOCX) [file pone.0312492.s012.docx]

**S1 Table:**

| Strain | Description | Light growth rate | Dark growth rate |
| --- | --- | --- | --- |
| SAWy119 | Parent | 0.241 ± 0.013 hr^-1^ | 0.207±0.001 hr^-1^ |
| SAWy518 | *Δhmg2* | 0.221 ± 0.173 hr^-1^ | 0.209±0.005 hr^-1^ |
| SAWy524 | optoMEV | 0.235 ± 0.008 hr^-1^ | 0.026±0.004 hr^-1^ |
