# Supplementary material for: Optogenetic screening of MCT1 activity implicates a cluster of non-steroidal anti-inflammatory drugs (NSAIDs) as inhibitors of lactate transport
Source: PLoS One. 2024 Dec 12;19(12):e0312492. doi: 10.1371/journal.pone.0312492 (PMC11637378; doi:10.1371/journal.pone.0312492)
Supplement: S2 Table — (DOCX) [file pone.0312492.s013.docx]

**S2 Table:**

| Strain | Description | Light growth rate | Dark growth rate |
| --- | --- | --- | --- |
| SAWy548 | EV | 0.256±0.005 hr^-1^ | 0.048±0.001 hr^-1^ |
| SAWy549 | MCT1(F360C) | 0.167±0.003 hr^-1^ | 0.042±0.001 hr^-1^ |
| SAWy550 | MCT1(F360C), CD147 | 0.138±0.016 hr^-1^ | 0.042±0.001 hr^-1^ |
| SAWy526 | MCT1(WT), CD147 | 0.205±0.007 hr^-1^ | 0.037±0.001 hr^-1^ |
| SAWy527 | MCT1(F360C), CD147-2 | 0.109±0.008 hr^-1^ | 0.057±0.001 hr^-1^ |
