# Supplementary material for: Optogenetic screening of MCT1 activity implicates a cluster of non-steroidal anti-inflammatory drugs (NSAIDs) as inhibitors of lactate transport
Source: PLoS One. 2024 Dec 12;19(12):e0312492. doi: 10.1371/journal.pone.0312492 (PMC11637378; doi:10.1371/journal.pone.0312492)
Supplement: S3 Table — (DOCX) [file pone.0312492.s014.docx]

**S3 Table:**

| Strain | Description | Growth rate |
| --- | --- | --- |
| By4741 | WT | 0.068 ± 0.003 hr^-1^ |
| SAWy575 | by4741 *Δjen1* empty vector | 0.062 ± 0.002 hr^-1^ |
| SAWy581 | by4741 *Δjen1* SUC2n-MCT1-GAP1c | 0.115 ± 0.001 hr^-1^ |
