# Supplementary material for: Optogenetic screening of MCT1 activity implicates a cluster of non-steroidal anti-inflammatory drugs (NSAIDs) as inhibitors of lactate transport
Source: PLoS One. 2024 Dec 12;19(12):e0312492. doi: 10.1371/journal.pone.0312492 (PMC11637378; doi:10.1371/journal.pone.0312492)
Supplement: S4 Table — (DOCX) [file pone.0312492.s015.docx]

**S4 Table:**

| Strain | Description | Dark growth rate |
| --- | --- | --- |
| SAWy623 | EV | 0.042 ± 0.003 hr^-1^ |
| SAWy530 | MCT1 | 0.048 ± 0.007 hr^-1^ |
| SAWy560 | SUC2n-MCT1-GAP1c, CD147-2 | 0.158 ± 0.006 hr^-1^ |
| SAWy570 | pTEF1_ SUC2n-MCT1-GAP1c, CD147-2 | 0.143 ± 0.027 hr^-1^ |
| SAWy571 | pTPI1_ SUC2n-MCT1-GAP1c, CD147-2 | 0.086 ± 0.007 hr^-1^ |
| SAWy572 | SUC2n-MCT1(WT)-GAP1c, CD147-2 | 0.034 ± 0.005 hr^-1^ |
| SAWy573 | SMD154Bn-MCT1-GAP1c, CD147-2 | 0.098 ± 0.010 hr^-1^ |
| SAWy528 | AFROPTn-MCT1-GAP1c, CD147-2 | 0.148 ± 0.002 hr^-1^ |
| SAWy529 | MCT1-GAP1c, CD147-2 | 0.081 ± 0.008 hr^-1^ |
