# Supplementary material for: Optogenetic screening of MCT1 activity implicates a cluster of non-steroidal anti-inflammatory drugs (NSAIDs) as inhibitors of lactate transport
Source: PLoS One. 2024 Dec 12;19(12):e0312492. doi: 10.1371/journal.pone.0312492 (PMC11637378; doi:10.1371/journal.pone.0312492)
Supplement: S5 Table — (DOCX) [file pone.0312492.s016.docx]

**S5 Table:**

| Strain | Description | Dark (10 mM MVA) | Dark (2 mM MVA) |
| --- | --- | --- | --- |
| SAWy623 | EV | 0.033±0.005 hr^-1^ | 0.027±0.004 hr^-1^ |
| SAWy574 | MCT1-JEN1c, CD147-2 | 0.165±0.015 hr^-1^ | 0.052±0.009 hr^-1^ |
| SAWy741 | JEN1n-MCT1, CD147-2 | 0.195±0.008 hr^-1^ | 0.116±0.011 hr^-1^ |
| SAWy669 | JEN1n-MCT1-JEN1c, CD147-2 | 0.178±0.008 hr^-1^ | 0.092±0.018 hr^-1^ |
