# Supplementary material for: Optogenetic screening of MCT1 activity implicates a cluster of non-steroidal anti-inflammatory drugs (NSAIDs) as inhibitors of lactate transport
Source: PLoS One. 2024 Dec 12;19(12):e0312492. doi: 10.1371/journal.pone.0312492 (PMC11637378; doi:10.1371/journal.pone.0312492)
Supplement: S6 Table — (DOCX) [file pone.0312492.s017.docx]

**S6 Table:**

| Strain | Description | Light | Dark |
| --- | --- | --- | --- |
| SAWy623 | EV DMSO | 0.282±0.011 hr^-1^ | 0.022±0.004 hr^-1^ |
| SAWy623 | EV 10 µM AZD3965 | 0.281±0.014 hr^-1^ | 0.027±0.002 hr^-1^ |
| SAWy741 | JEN1n-MCT1 DMSO | 0.282±0.007 hr^-1^ | 0.145±0.009 hr^-1^ |
| SAWy741 | JEN1n-MCT1 10 µM AZD3965 | 0.281±0.001 hr^-1^ | 0.030±0.003 hr^-1^ |
