# Supplementary material for: Optogenetic screening of MCT1 activity implicates a cluster of non-steroidal anti-inflammatory drugs (NSAIDs) as inhibitors of lactate transport
Source: PLoS One. 2024 Dec 12;19(12):e0312492. doi: 10.1371/journal.pone.0312492 (PMC11637378; doi:10.1371/journal.pone.0312492)
Supplement: S7 Table — (DOCX) [file pone.0312492.s018.docx]

**S7 Table:**

| **Plasmid** | **Contents** | **Source** |
| --- | --- | --- |
| p20 | 2µ *URA3* empty vector | [1] |
| pYZ125 | CEN/ARS *URA3* empty vector | [1] |
| SAWlig399 | 2µ URA3 P_GPD1__MCT1(F360C)_T_CYC1_ |  |
| SAWlig400 | CEN/ARS URA3 P_GPD1__MCT1(F360C)_T_CYC1_ |  |
| SAWlig401 | 2µ URA3 P_GPD1__MCT1(F360C)_T_CYC1,_ P_PGK1__CD147_T_ADH1_ |  |
| SAWlig419 | 2µ URA3 P_GPD1__MCT1(WT)_T_CYC1,_ P_PGK1__CD147_T_ADH1_ |  |
| SAWlig420 | 2µ URA3 P_GPD1__MCT1(F360C)-GFP_T_CYC1_ |  |
| SAWlig421 | 2µ URA3 P_GPD1__MCT1(F360C)-GFP-GAP1c_T_CYC1_ |  |
| SAWlig422 | 2µ URA3 P_GPD1__MCT1(F360C)-GFP-JEN1c_T_CYC1_ |  |
| SAWlig433 | 2µ URA3 P_GPD1__MCT1(F360C)-GAP1c_T_CYC1_ |  |
| SAWlig434 | 2µ URA3 P_GPD1__MCT1(F360C)-JEN1c_T_CYC1_ |  |
| SAWlig435 | 2µ URA3 P_GPD1__MCT1(F360C)-HXT6c_T_CYC1_ |  |
| SAWlig436 | 2µ URA3 P_GDP1__MCT1(F360C) -PDR5c_T_CYC1_ |  |
| SAWlig438 | 2µ URA3 P_GPD1__SUC2n-MCT1(F360C)_T_CYC1_ |  |
| SAWlig440 | 2µ URA3 P_GPD1__SUC2n-MCT1(F360C)-GFP_T_CYC1_ |  |
| SAWlig444 | 2µ URA3 P_GDP1__MCT1(F360C)-GFP-HXT6c_T_CYC1_ |  |
| SAWlig445 | 2µ URA3 P_GDP1__MCT1(F360C)-GFP-PDR5c_T_CYC1_ |  |
| SAWlig447 | 2µ URA3 P_GPD1__MCT1(F360C)_T_CYC1,_ P_PGK1__CD147-2_T_ADH1_ |  |
| SAWlig460 | 2µ URA3 P_GDP1__SUC2n-MCT1(F360C)-GFP-GAP1c_T_CYC1_ |  |
| SAWlig462 | 2µ URA3 P_GDP1__SUC2n-MCT1-GAP1c_T_CYC1_ |  |
| SAWlig465 | CEN/ARS URA3 P_TEF1__ SUC2n-MCT1(F360C)-GAP1c_T_CYC1_, P_PGK1__CD147-2_T_ADH1_ |  |
| SAWlig466 | CEN/ARS URA3 P_TPI1__ SUC2n-MCT1(F360C)-GAP1c_T_CYC1_, P_PGK1__CD147-2_T_ADH1_ |  |
| SAWlig467 | CEN/ARS URA3 P_GDP1__SUC2n-MCT1(WT)-GAP1c_T_CYC1_, P_PGK1__CD147-2_T_ADH1_ |  |
| SAWlig468 | CEN/ARS URA3 P_GDP1__SMD154Bn-MCT1(F360C)-GAP1c_T_CYC1_, P_PGK1__CD147-2_T_ADH1_ |  |
| SAWlig469 | CEN/ARS URA3 P_GPD1__AFROPTn-MCT1(F360C)-GAP1c_T_CYC1_, P_PGK1__CD147-2_T_ADH1_ |  |
| SAWlig484 | CEN/ARS URA3 P_GPD1__SUC2n-MCT1(F360C)-GAP1c_T_CYC1_, P_PGK1__CD147-2_T_ADH1_ |  |
| SAWlig488 | CEN/ARS URA3 P_GDP1__MCT1(F360C)-GFP_T_CYC1_ |  |
| SAWlig489 | CEN/ARS URA3 P_GDP1__SUC2n-MCT1(F360C)-GFP-GAP1c_T_CYC1_ |  |
| SAWlig529 | CEN/ARS URA3 P_GDP1__JEN1n15-MCT1(F360C)-JEN1c431_T_CYC1_, P_PGK1__CD147-2_T_ADH1_ |  |
| SAWlig530 | CEN/ARS URA3 P_GDP1__JEN1n15-MCT1(F360C)-JEN1c439_T_CYC1_, P_PGK1__CD147-2_T_ADH1_ |  |
| SAWlig531 | CEN/ARS URA3 P_GDP1__JEN1n15-MCT1(F360C)-JEN1c461_T_CYC1_, P_PGK1__CD147-2_T_ADH1_ |  |
| SAWlig532 | CEN/ARS URA3 P_GDP1__JEN1n15-MCT1(F360C)-JEN1c_T_CYC1_, P_PGK1__CD147-2_T_ADH1_ |  |
| SAWlig533 | CEN/ARS URA3 P_GDP1__JEN1n-MCT1(F360C)-JEN1c431_T_CYC1_, P_PGK1__CD147-2_T_ADH1_ |  |
| SAWlig534 | CEN/ARS URA3 P_GDP1__JEN1n-MCT1(F360C)-JEN1c439_T_CYC1_, P_PGK1__CD147-2_T_ADH1_ |  |
| SAWlig535 | CEN/ARS URA3 P_GDP1__JEN1n-MCT1(F360C)-JEN1c461_T_CYC1_, P_PGK1__CD147-2_T_ADH1_ |  |
| SAWlig536 | CEN/ARS URA3 P_GDP1__JEN1n-MCT1(F360C)-JEN1c_T_CYC1_, P_PGK1__CD147-2_T_ADH1_ |  |
| SAWlig574 | CEN/ARS URA3 P_GDP1__MCT1(F360C)-GAP1c_T_CYC1_, P_PGK1__CD147-2_T_ADH1_ |  |
| SAWlig577 | CEN/ARS URA3 P_GDP1__MCT1(F360C)_JEN1c_T_CYC1_, P_PGK1__CD147-2_T_ADH1_ |  |
| SAWlig578 | CEN/ARS URA3 P_GDP1__JEN1n-MCT1(F360C) _T_CYC1_, P_PGK1__CD147-2_T_ADH1_ |  |
| SAWlig578_wt | CEN/ARS URA3 P_GDP1__JEN1n-MCT1(WT) _T_CYC1_, P_PGK1__CD147-2_T_ADH1_ |  |

1. Zhao EM, Zhang Y, Mehl J, et al. Optogenetic regulation of engineered cellular metabolism for microbial chemical production. *Nature 2018 555:7698*. 2018;555(7698):683-687. doi:10.1038/nature26141
