# Supplementary material for: Optogenetic screening of MCT1 activity implicates a cluster of non-steroidal anti-inflammatory drugs (NSAIDs) as inhibitors of lactate transport
Source: PLoS One. 2024 Dec 12;19(12):e0312492. doi: 10.1371/journal.pone.0312492 (PMC11637378; doi:10.1371/journal.pone.0312492)
Supplement: S8 Table — (DOCX) [file pone.0312492.s019.docx]

**S8 Table:**

| **Strain Name** | **Genotype** | **Source** |
| --- | --- | --- |
| by4741 | MATa his3Δ1 leu2Δ0 met15Δ0 ura3Δ0 | [1] |
| by4741 jen1Δ | MATa his3Δ1 leu2Δ0 met15Δ0 ura3Δ0 jen1Δ::G418 | [1] |
| CEN.PK2-1C | MATa his3Δ1 leu2-3_112 trp1-289 ura3-53 | [2] |
| yTK29 | CENPK2-1C TRP1::P_TPI1__SS-mCherry-HDEL_T_CYC1_ | [3] |
| SAWy119 | CENPK2-1C *gal80∆*, *gal4∆*::HygB *his3*::*HIS3*, P_TEF1__VP16-EL222_T_CYC1_, P_C120__GAL80_T_ADH1_, P_ADH1__GAL4_T_ACT1_, P_C120__GAL80_T_ADH1_ | [4] |
| SAWy518 | SAWy119 *hmg2∆*::neoMX | [4] |
| SAWy524 | SAWy518 pC120::HMG1 | [4] |
| SAWy526 | SAWy524 2µ URA3 P_GPD1__MCT1(WT)_T_CYC1,_ P_PGK1__CD147_T_ADH1_ (SAWlig419) |  |
| SAWy527 | SAWy524 2µ URA3 P_GPD1__MCT1(F360C)_T_CYC1,_ P_PGK1__CD147-2_T_ADH1_ (SAWlig447) |  |
| SAWy528 | SAWy524 CEN/ARS URA3 P_GPD1__AFROPTn-MCT1(F360C)-GAP1c_T_CYC1_, P_PGK1__CD147-2_T_ADH1_ (SAWlig469) |  |
| SAWy529 | SAWy524 CEN/ARS URA3 P_GDP1__MCT1(F360C)-GAP1c_T_CYC1_, P_PGK1__CD147-2_T_ADH1_ (SAWlig574) |  |
| SAWy530 | SAWy524 CEN/ARS URA3 P_GPD1__MCT1(F360C)_T_CYC1_ (SAWlig400) |  |
| SAWy548 | SAWy524 2µ *URA3* empty vector (p20) |  |
| SAWy549 | SAWy524 2µ URA3 P_GPD1__MCT1(F360C)_T_CYC1_ (SAWlig399) |  |
| SAWy550 | SAWy524 2µ URA3 P_GPD1__MCT1(F360C)_T_CYC1,_ P_PGK1__CD147_T_ADH1_ (SAWlig401) |  |
| SAWy560 | SAWy524 CEN/ARS URA3 P_GPD1__SUC2n-MCT1(F360C)-GAP1c_T_CYC1_, P_PGK1__CD147-2_T_ADH1_ (SAWlig484) |  |
| SAWy570 | SAWy524 CEN/ARS URA3 P_TEF1__ SUC2n-MCT1(F360C)-GAP1c_T_CYC1_, P_PGK1__CD147-2_T_ADH1_ (SAWlig465) |  |
| SAWy571 | SAWy524 CEN/ARS URA3 P_TPI1__ SUC2n-MCT1(F360C)-GAP1c_T_CYC1_, P_PGK1__CD147-2_T_ADH1_ (SAWlig466) |  |
| SAWy572 | SAWy524 CEN/ARS URA3 P_GDP1__SUC2n-MCT1(WT)-GAP1c_T_CYC1_, P_PGK1__CD147-2_T_ADH1_ (SAWlig467) |  |
| SAWy573 | SAWy524 CEN/ARS URA3 P_GDP1__SMD154Bn-MCT1(F360C)-GAP1c_T_CYC1_, P_PGK1__CD147-2_T_ADH1_ (SAWlig468) |  |
| SAWy574 | SAWy524 CEN/ARS URA3 P_GDP1__MCT1(F360C)_JEN1c_T_CYC1_, P_PGK1__CD147-2_T_ADH1_ (SAWlig577) |  |
| SAWy575 | by4741 jen1Δ 2µ *URA3* empty vector (p20) |  |
| SAWy576 | by4741 jen1Δ 2µ URA3 P_GPD1__MCT1(F360C)_T_CYC1_ (SAWlig399) |  |
| SAWy577 | by4741 jen1Δ 2µ URA3 P_GPD1__MCT1(F360C)-GAP1c_T_CYC1_ (SAWlig433) |  |
| SAWy578 | by4741 jen1Δ 2µ URA3 P_GPD1__MCT1(F360C)-JEN1c_T_CYC1_ (SAWlig434) |  |
| SAWy579 | by4741 jen1Δ 2µ URA3 P_GPD1__MCT1(F360C)-HXT6c_T_CYC1_ (SAWlig435) |  |
| SAWy580 | by4741 jen1Δ 2µ URA3 P_GPD1__SUC2n-MCT1(F360C)_T_CYC1_ (SAWlig438) |  |
| SAWy581 | by4741 jen1Δ 2µ URA3 P_GDP1__SUC2n-MCT1-GAP1c_T_CYC1_ (SAWlig462) |  |
| SAWy582 | yTK29 2µ URA3 P_GPD1__MCT1(F360C)-GFP_T_CYC1_ (SAWlig420) |  |
| SAWy583 | yTK29 2µ URA3 P_GPD1__SUC2n-MCT1(F360C)-GFP_T_CYC1_ (SAWlig440) |  |
| SAWy584 | yTK29 2µ URA3 P_GDP1__SUC2n-MCT1(F360C)-GFP-GAP1c_T_CYC1_ (SAWlig460) |  |
| SAWy585 | yTK29 CEN/ARS URA3 P_GDP1__MCT1(F360C)-GFP_T_CYC1_ (SAWlig488) |  |
| SAWy586 | yTK29 CEN/ARS URA3 P_GDP1__SUC2n-MCT1(F360C)-GFP-GAP1c_T_CYC1_ (SAWlig489) |  |
| SAWy587 | yTK29 2µ URA3 P_GPD1__MCT1(F360C)-GFP-GAP1c_T_CYC1_ (SAWlig421) |  |
| SAWy592 | yTK29 2µ URA3 P_GPD1__MCT1(F360C)-GFP-JEN1c_T_CYC1_ (SAWlig422) |  |
| SAWy593 | yTK29 2µ URA3 P_GDP1__MCT1(F360C)-GFP-HXT6c_T_CYC1_ (SAWlig444) |  |
| SAWy594 | yTK29 2µ URA3 P_GDP1__MCT1(F360C)-GFP-PDR5c_T_CYC1_ (SAWlig445) |  |
| SAWy595 | by4741 jen1Δ 2µ URA3 P_GDP1__MCT1(F360C) -PDR5c_T_CYC1_ (SAWlig436) |  |
| SAWy623 | SAWy524 CEN/ARS *URA3* empty vector (pYZ125) |  |
| SAWy662 | SAWy524 CEN/ARS URA3 P_GDP1__JEN1n15-MCT1(F360C)-JEN1c431_T_CYC1_, P_PGK1__CD147-2_T_ADH1_ (SAWlig529) |  |
| SAWy663 | SAWy524 CEN/ARS URA3 P_GDP1__JEN1n15-MCT1(F360C)-JEN1c439_T_CYC1_, P_PGK1__CD147-2_T_ADH1_ (SAWlig530) |  |
| SAWy664 | SAWy524 CEN/ARS URA3 P_GDP1__JEN1n15-MCT1(F360C)-JEN1c461_T_CYC1_, P_PGK1__CD147-2_T_ADH1_ (SAWlig531) |  |
| SAWy665 | SAWy524 CEN/ARS URA3 P_GDP1__JEN1n15-MCT1(F360C)-JEN1c_T_CYC1_, P_PGK1__CD147-2_T_ADH1_ (SAWlig532) |  |
| SAWy666 | SAWy524 CEN/ARS URA3 P_GDP1__JEN1n-MCT1(F360C)-JEN1c431_T_CYC1_, P_PGK1__CD147-2_T_ADH1_ (SAWlig533) |  |
| SAWy667 | SAWy524 CEN/ARS URA3 P_GDP1__JEN1n-MCT1(F360C)-JEN1c439_T_CYC1_, P_PGK1__CD147-2_T_ADH1_ (SAWlig534) |  |
| SAWy668 | SAWy524 CEN/ARS URA3 P_GDP1__JEN1n-MCT1(F360C)-JEN1c461_T_CYC1_, P_PGK1__CD147-2_T_ADH1_ (SAWlig535) |  |
| SAWy669 | SAWy524 CEN/ARS URA3 P_GDP1__JEN1n-MCT1(F360C)-JEN1c_T_CYC1_, P_PGK1__CD147-2_T_ADH1_ (SAWlig536) |  |
| SAWy741 | SAWy524 CEN/ARS URA3 P_GDP1__JEN1n-MCT1(F360C) _T_CYC1_, P_PGK1__CD147-2_T_ADH1_ (SAWlig578) |  |
| SAWy741_wt | SAWy524 CEN/ARS URA3 P_GDP1__JEN1n-MCT1(WT) _T_CYC1_, P_PGK1__CD147-2_T_ADH1_ (SAWlig578_wt) |  |

1. Giaever G, Chu AM, Ni L, et al. Functional profiling of the Saccharomyces cerevisiae genome. *Nature*. 2002;418(6896):387-391. doi:10.1038/NATURE00935
2. Entian KD, Kötter P. 25 Yeast Genetic Strain and Plasmid Collections. *Methods in Microbiology*. 2007;36:629-666. doi:10.1016/S0580-9517(06)36025-4
3. Kichuk T, Dhamankar S, Malani S, et al. Article Using MitER for 3D analysis of mitochondrial morphology and ER contacts. *Cell Reports Methods*. 2024;4:100692. doi:10.1016/j.crmeth.2023.100692
4. Wegner SA, Jiang V, Cortez JD, Avalos JL. [In preparation].
